# Fluorescent labeling of biocompatible block copolymers: synthetic strategies and applications in bioimaging

**DOI:** 10.1039/d1ma00110h

**Published:** 2021-04-06

**Authors:** Sophie Bou, Andrey S. Klymchenko, Mayeul Collot

**Affiliations:** Laboratoire de Bioimagerie et Pathologies, UMR 7021, CNRS/Université de Strasbourg 74 route du Rhin 67401 Illkirch-Graffenstaden France mayeul.collot@unistra.fr

## Abstract

Among biocompatible materials, block copolymers (BCPs) possess several advantages due to the control of their chemistry and the possibility of combining various blocks with defined properties. Consequently, BCPs drew considerable attention as biocompatible materials in the fields of drug delivery, medicine and bioimaging. Fluorescent labeling of BCPs quickly appeared to be a method of choice to image and track these materials in order to better understand the nature of their interactions with biological media. However, incorporating fluorescent markers (FM) into BCPs can appear tricky; we thus intend to help chemists in this endeavor by reviewing recent advances made in the last 10 years. With the choice of the FM being of prior importance, we first reviewed their photophysical properties and functionalities for optimal labeling and imaging. In the second part the different chemical approaches that have been used in the literature to fluorescently label BCPs have been reviewed. We also report and discuss relevant applications of fluorescent BCPs in bioimaging.

## Abstract 2

This review describes the different chemical approaches and strategies to fluorescently label block copolymers. The review also focuses on the properties of fluorescent markers and the bioimaging applications allowed by the labeling.

## Graphical abstract

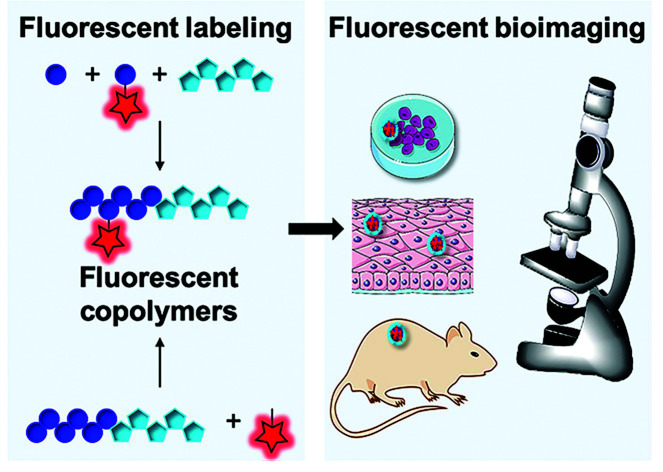

## Introduction

Biocompatible polymers are considered as materials that do not interfere with biological environments.^1^ This appealing feature was widely demonstrated by *in vitro* and *in vivo* assays providing evidence of their non-toxic behavior. Some of these biocompatible polymers have been already approved for use in humans by drug regulation agencies such as the FDA (Food and Drug Administration), EMA (European Medicines Agency) or CFDA (China Food and Drug Administration).^2^ Due to their different properties including water-solubility,^3^ drug carriers,^4^ pH and thermo-sensitivity,^5,6^ biocompatible polymers have recently drawn considerable attention in the fields of materials,^7^ medicine,^8^ bioimaging,^9,10^ and drug delivery^11^ and also as a new class of medicine support.^12^ Among biocompatible polymers, biopolymers like polysaccharides,^13,14^ polypeptides,^15^ and polynucleotides^16^ possess important advantages including biodegradability and a negligible impact on the environment. However, their biosynthesis process and tedious synthesis limit the design of new biomaterials with desired properties. Therefore, new synthetic and biocompatible polymers were developed in order to better control their size and to finely tune their properties including degradation kinetics,^17^ amphiphilicity,^18^ or drug release control.^19^ Among synthetic biocompatible polymers, block copolymers (BCPs) are considered as promising materials as they present several advantages due to the possible combination of various blocks.^20^ Indeed, BCPs are composed of two or more blocks with specific physico-chemical properties or functionalities that can be combined, thus leading to materials with new properties.^21^ BCPs often comprise at least one hydrophobic block and at least one hydrophilic block leading to amphiphilic polymers. This property is mainly used in the preparation of nanoparticles (NPs) as the solvophobic (*e.g.* hydrophobic) interaction ensures a core–shell structure.^22^ Moreover, the combination of multiple blocks (diblock, triblock or more) may expand the range of properties of the final material and thus increase the versatility of BCPs compared to biopolymers. Indeed, the control of the properties of BCPs was a considerable achievement, which allowed their use in drug release or bioimaging applications.^23,24^ Although the biocompatibility of BCPs can be ensured, understanding of their *in vivo* interactions is essential. In order to decipher the nature of their interactions in biological media and their biodistribution, the fluorescent labeling of BCPs was often considered. Indeed, fluorescence spectroscopy and microscopy techniques have proven to be powerful tools to study biological mechanisms,^25^ interactions between medical devices and tissues^26^ and drug release.^27^ Fluorescent labeling also allows tracking of copolymers *in cellulo* and *in vivo* with high temporal and spatial resolution.^12,28^ Moreover, fluorescently labeled BCPs can potentially combine their imaging and drug release properties, thus acquiring the ability to act as theranostic agents.^29^

All these applications rely on the strategic choice of fluorescent markers (FMs) for labeling the BCP of interest. Consequently, the incorporation of a FM into a BCP remains a key step, where two main methods can be considered: physical entrapment^30^ and chemical ligation.^31^ Although the first approach consisting of encapsulating the FM within the polymer matrix was proven to be very efficient in some cases,^32^ it can present a main drawback: the leakage of the FM out of the polymer which can lead to biased conclusions in bioimaging.^33^ To circumvent this phenomenon and its undesirable consequences, the covalent binding of the FM to the polymer is a robust alternative. Previous reviews addressed the fluorescent labeling of polymers showing the real interest in this research area for bioimaging.^2,7^ Herein, we focused on the fluorescent labeling of biocompatible BCPs through a review of different chemical approaches and strategies. In the first part, we reported on fluorophores (FMs) that were used, and their properties and advantages were discussed. In the second part the chemical modifications of BCPs and approaches to obtain fluorescently labeled BCPs were reviewed. Some examples are detailed to show the implications of fluorescent BCPs in bioimaging.

## 1. Fluorophores

The fluorescent labeling of BCPs involves the selection of a FM that can be driven by several criteria. First, the fluorophore should possess a functionality allowing its covalent ligation to the monomer or the polymer. The involved chemistry should be efficient and should not require harsh conditions (high temperature, presence of strong acids or bases) that could be detrimental to the fluorophore itself but could also lead to partial destruction of the polymer. Although it is sometimes difficult to find fluorescent markers with adapted reactive groups, numerous FMs have been developed and are now commercially available with a wide choice of functionalities, the most common ones being free amine (–NH_2_) as well as amine reactive groups, including carboxylate (–COOH), isothiocyanate (–NCS) and active ester like *N*-hydroxysuccinimide (NHS). Functionalities adapted to click chemistry including maleimide and thiol (–SH) for hetero-Michael reaction^34^ and alkyne/azide (–N_3_) for Huisgen cycloaddition^35^ are also widely proposed. The second criterion is the physical properties of the fluorophore. As the FM can dramatically modify the properties of the BCP, its hydrophobicity, size and global charge should also be taken into account. Although properties such as polarity or hydrophobicity are rarely considered given the relatively small amount of FM required for labeling, it should be kept in mind that it might modify the physico-chemical properties of the studied BCP to some extent. For instance, cationic FMs like cyanines, rhodamines or benzopyrylium (*vide infra*) might considerably modify the charge surface of the obtained polymeric NPs and thus the cell penetration ability or the biodistribution, retention and elimination in *in vivo* studies. The water solubility and aqueous stability of FMs are generally not considered in block copolymer labeling mostly because the chemistry of labeling generally occurs in organic solvents and also because, once embedded in the polymeric NPs, the dye is overall preserved from aqueous environments. Importantly, photophysical properties such as the absorption and emission maxima, brightness (extinction coefficient expressed in M^−1^ cm^−1^ and quantum yields *Φ*) and broadness of the bands are of prior importance as they must be adapted to the microscope or fluorescence imaging setting and the modalities used during imaging. Once again, commercially available fluorophores offer a wide choice of photophysical properties with excitation and emission spectra spanning from the UV-visible to the near-infrared (NIR) range, allowing optimized compatibility with defined excitation sources and emission filters of the imaging setup for various applications. For *in vivo* applications, fluorescent materials emitting in the NIR range are better suited. Although fluorophores emitting in the visible range can be used, the tissues interfere with the excitation and emission light in the UV-visible region due to the scattering and absorption by the species in the biological media, mainly hemoglobin (Hb), and its oxidized form (HbO_2_).^36^ Furthermore, the tissues display high auto-fluorescence in this region,^37^ thus increasing difficulties in imaging. The tissues possess two transparency windows in the NIR region, where the absorption, auto-fluorescence and light scattering are reduced. These regions, allowing in-depth imaging in real time, are 650–950 nm for NIR-I^38^ and 950–1350 nm for NIR-II.^39^

More complex systems can also be developed using a pair of dyes allowing the Förster Resonance Energy Transfer (FRET) phenomenon.^40,41^ FRET is a photophysical process which consists in an excitation of the donor dye, followed by an energy transfer to the acceptor dye, resulting in the emission of the latter. The phenomenon is possible if the emission spectrum of the donor dye overlaps with the absorption spectrum of the acceptor dye. Furthermore, the donor and acceptor fluorophores must be spatially close to each other (1–10 nm). Consequently, FRET has been widely used in biology and bioimaging for tracking close species or assessing drug release.^42^ The incorporation of a FRET-pair into a polymer allows its fluorescent tracking, but the monitoring of the FRET signal can also provide information about its degradation kinetics, drug release kinetics, as well as the fate of degraded species.^43^

Finally, the toxicity of the FM is a criterion that is often not considered. Although the NPs resulting from fluorescent polymers generally display rather low *in vitro* cytotoxicity,^44–50^ the new physico-chemical properties of the labeled BCP can modify its retention and elimination profiles which could lead to *in vivo* toxic effects. Overall, the choice of the FM will lead to modifications of the physical and photophysical properties of the BCP and can also determine the applications in bioimaging.

We herein classified the reported fluorophores for BCP labeling into different families. For convenience, the reactive functions that served to label the copolymers are highlighted in red in the following figures. The maximum absorption and emission wavelengths of the corresponding fluorescently labeled copolymers in a defined solvent are also indicated.

### 1.1 Carbocyanines

**Fig. 1:**
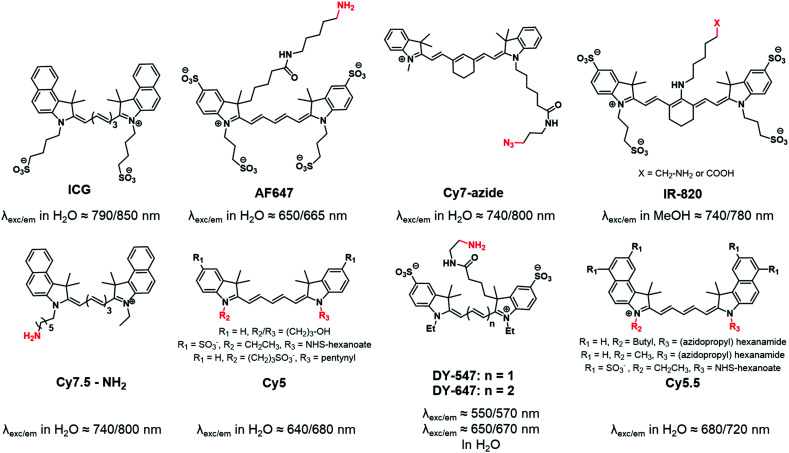
ICG and various carbocyanines that were used for fluorescent labeling of BCPs.

Carbocyanines are fluorophores displaying a positive charge delocalized through a conjugated polymethine chain. They display extremely high extinction coefficients (up to 250 000 M^−1^ cm^−1^) and can display high quantum yields in organic solvents or non-polar environments, thus making them one of the brightest families of dyes.^51^ Among carbocyanines, indocyanine green (ICG, Fig. 1) is a NIR emitting sulfonated cyanine 7.5 used in medical applications.^52^ Due to its low toxicity and rapid elimination, it was approved by the FDA and is now widely and successfully used for *in vivo* imaging. ICG is a typical example of a fluorophore displaying appealing photophysical properties but it cannot be used for copolymer labeling by covalent linkage due to the absence of a reactive group. Consequently, chemists developed Cy7.5 possessing similar photophysical properties and bearing a reactive functional group.^53^ It is noteworthy that the photophysical properties of cyanines can be tuned by modifying the conjugated backbone. For instance, the shortening of the polymethine chain (*n* in Fig. 1) or the removal of the fused benzene ring on the indolenine moiety leads to shorter delocalized electronic systems and thus to a significant hypsochromic effect (blue-shift).^54^ Functional moieties have been successfully added to the indolenine moiety of carbocyanines either by its *N*-alkylation (Fig. 1: Cy5, Cy5.5, Cy7-azide) or by the replacement of a geminal methyl group by a functionalizable chain (Fig. 1: AF647, IR-820). The wide variety of reactive functions such as alkyne,^55^ azide,^56^ amine,^50,51^ hydroxyl,^59^ carboxylic acid^50^ or *N*-hydroxysuccinimide (NHS)^60^ ensures the versatility of fluorescent labeling and most of the cyanines displaying these functions are now commercially available.^61,62^ However, one should note that some carbocyanines, especially Cy7 ones, may be chemically unstable.^63^

### 1.2. Benzopyrylium dyes

**Fig. 2:**
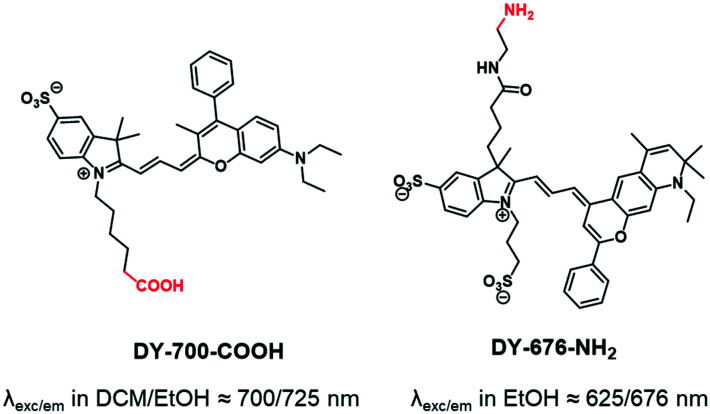
Benzopyrylium DY-700 and DY-676 used to label BCPs.

Whereas carbocyanine possesses a positive charge delocalized on two nitrogens, the charge of benzopyrylium is delocalized between a nitrogen and an oxygen (Fig. 2). Like carbocyanines, benzopyrylium dyes possess high brightness combined with long absorption and emission wavelengths, which are advantageous features for *in vivo* imaging applications. Moreover, the same modifications on the indoleninium moiety were performed on benzopyrylium dyes to make them functionalizable. DY-676 and DY-700 are commercially available and functionalizable fluorophores developed by Dyomics^64^ and were successfully used for labeling of copolymers through a carboxylate function^49^ or an amino-derivative,^65^ respectively.

### 1.3. Push–pull dyes

**Fig. 3:**
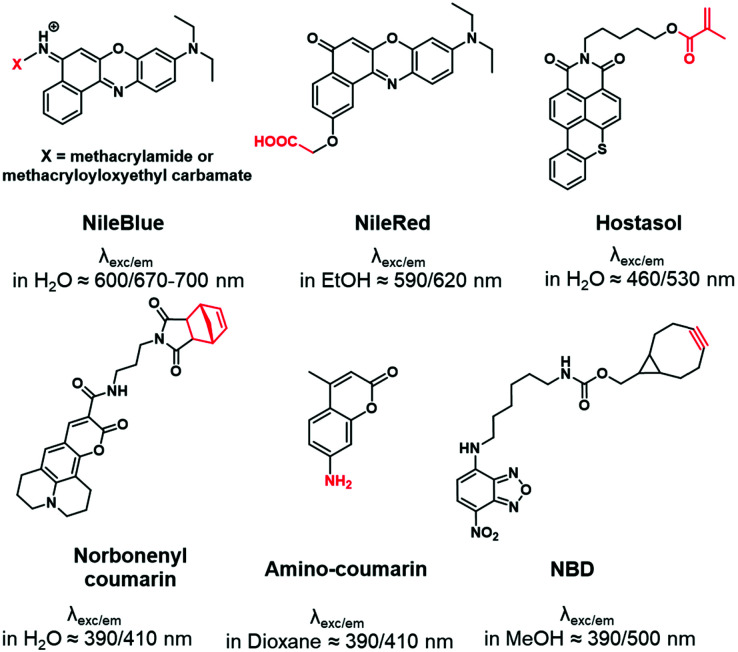
Structures of functionalizable push–pull dyes used for BCP labeling.

“Push–pull” dyes like coumarins and Nile Red are characterized by the presence of an electron donor moiety connected to an electron withdrawing group through a conjugated backbone.^66^ This structure ensures that, after light absorption, the charge is transferred from the donor group to the acceptor, which creates a highly dipolar excited state.^67^ The latter relaxes depending on the interactions with the dipoles of the solvent leading to solvatochromic properties. In other words, their emissions depend on the nature of solvents where a red-shift is observed in polar solvents, while a blue-shift corresponds to non-polar solvents. This environmental sensitivity can be an advantage in bioimaging applications, allowing the determination of the polarity of the local environment or cellular compartments (lipid droplets, membranes, cytosol).^68,69^ In addition, their structure allows functionalization with different moieties such as alkyne,^70^ amine,^71^ carboxylic acid^72^ or even methacrylate,^73^ carbamate^74^ and norbonenyl^75^ (Fig. 3). Overall these fluorophores are chemically stable.

### 1.4. Xanthene dyes

**Fig. 4:**
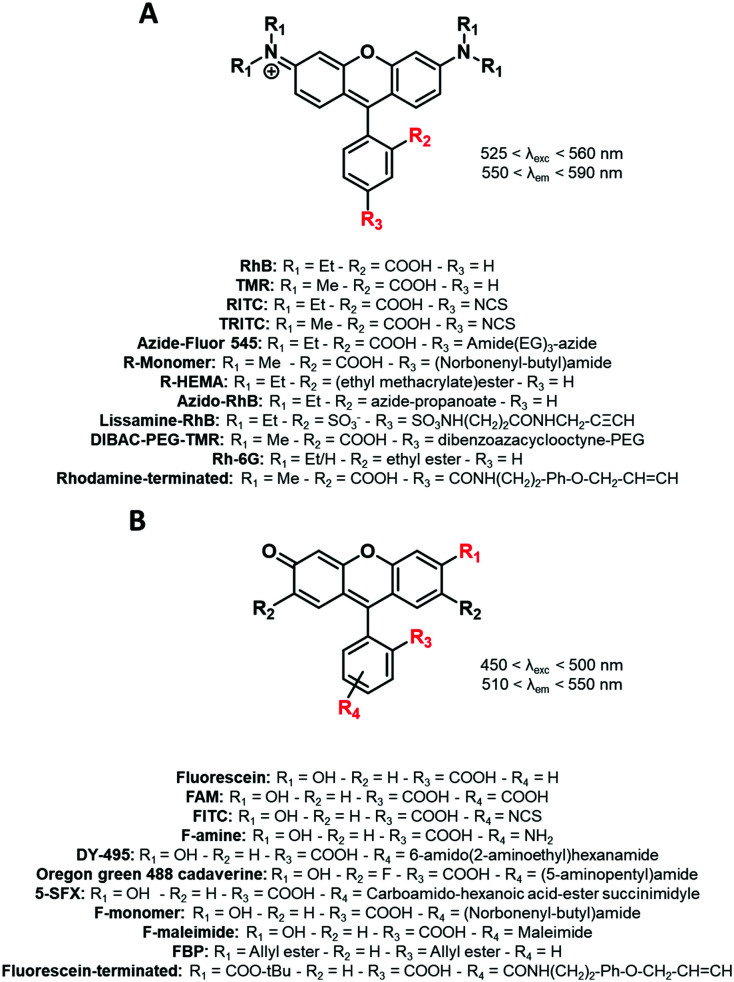
The xanthene FM used for the labeling of BCPs: (A) rhodamine and (B) fluorescein derivatives.

The popularity of xanthene dyes can be attributed to their high chemical stability and brightness (high extinction coefficients and quantum yields).^76^ In this family, positively charged rhodamine (Fig. 4A) and negatively charged fluorescein (Fig. 4B) which emit in the red and green regions, respectively, are the most famous and commonly used dyes (Fig. 4). Their functionalization was widely developed,^77,78^ giving access to numerous derivatives including NHS (*N*-hydroxysuccinimide) esters,^79^ azides,^80,81^ isothiocyanates (NCS),^71,82^ maleimides^83^ and more, explaining why these two fluorophores are highly represented in the fluorescent labeling of BCPs. It is however noteworthy that fluorescein derivatives (Fig. 4B), despite their extensive use, present some limitations such as low photostability and strong pH-dependency.^84^ Xanthene dyes are generally resistant to harsh conditions of synthesis.

### 1.5. Difluoroboron complexes

**Fig. 5:**
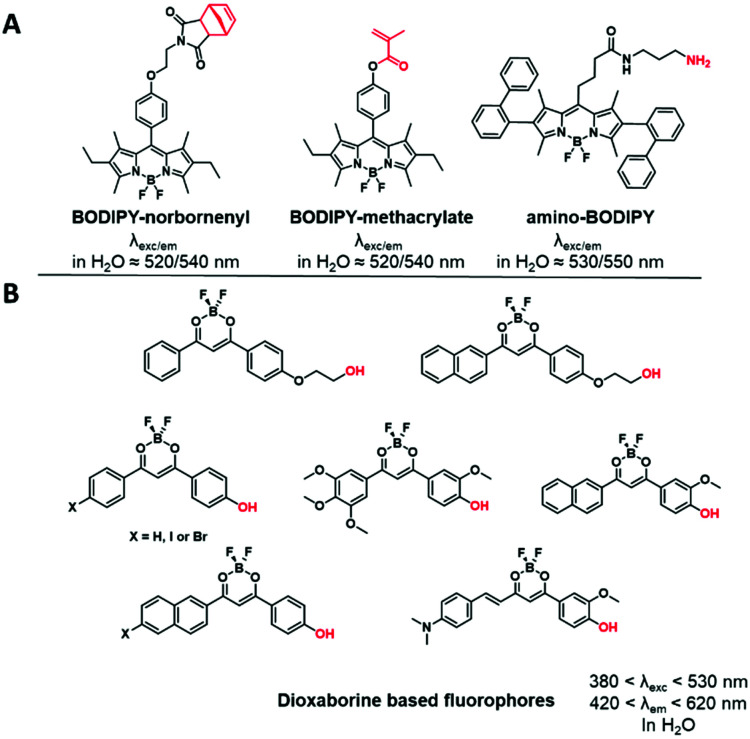
Examples of difluoroboron complexes used for BCP labeling: (A) BODIPY derivatives and (B) dioxaborine based fluorophores.

The members of this family contain a difluoroboron complex, which gives them an important brightness and enhanced photostability. The widely used BODIPY dyes present specific photophysical properties such as high extinction coefficients and quantum yields with narrow excitation and emission bands.^85^ Although their chemical modification was extensively explored providing a wide range of tunable emission colors,^86^ only yellow emitting BODIPYs were used for the labeling of BCPs by the introduction of reactive functions such as methacrylate,^48,87,88^ norbonenyl moiety^75^ and an amino-terminated linker on the phenyl ring at the *meso* position (Fig. 5).^89^ Similarly, dioxaborine-based fluorophores are composed of a BF_2_ moiety complexed by oxygens and are emerging as versatile fluorescent materials.^90^ Various derivatives were developed for BCP labeling using phenolic groups as connectors.^91,92^ It is noteworthy that the difluoroboron complex can give an electrophilic character to the fluorophore and thus impair its chemical stability in aqueous media.^93^

### 1.6. AIEgens

**Fig. 6:**
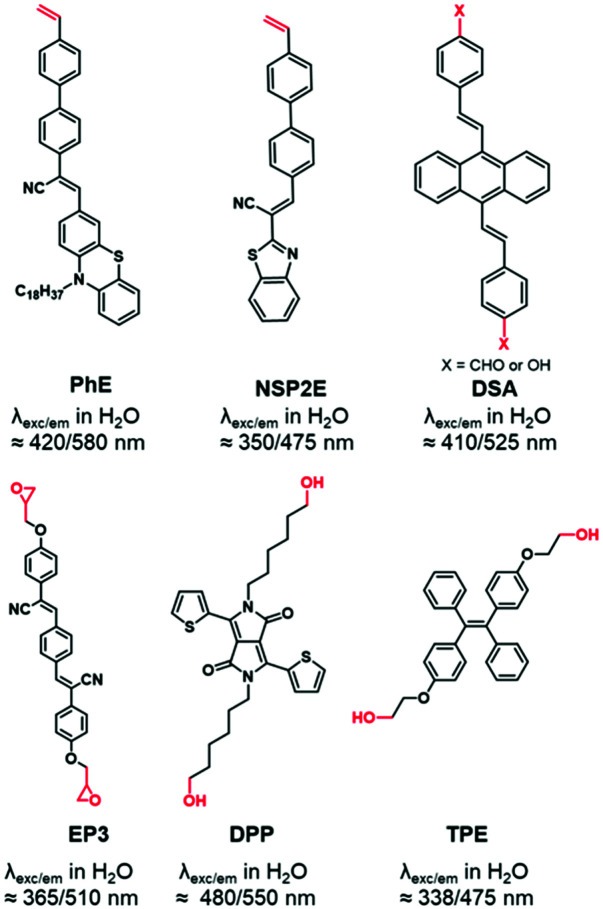
AIEgens displaying reactive functions used for BCP labeling.

AIEgens are chromophores that are poorly emissive in solution but highly emissive in the aggregated state.^94^ This “turn-on” phenomenon is driven by the restriction of intramolecular motion (RIM), including the restriction of intramolecular rotations and vibrations, which is described as aggregation induced emission (AIE). Substantial numbers of AIEgens with high quantum yields were developed with emission colors ranging from the visible to the NIR range.^95^ In contrast to other FMs, AIEgens do not suffer from aggregation caused quenching (ACQ) when loaded in high amounts into particles which is an obvious advantage.^96^ The labeling of BCPs was performed using AIEgens functionalized with a polymerizable styrene function,^44,45,97,98^ a benzaldehyde,^99^ an epoxide^100^ or hydroxyl groups^101,102^ (Fig. 6).

### 1.7. NIR-II dyes

**Fig. 7:**
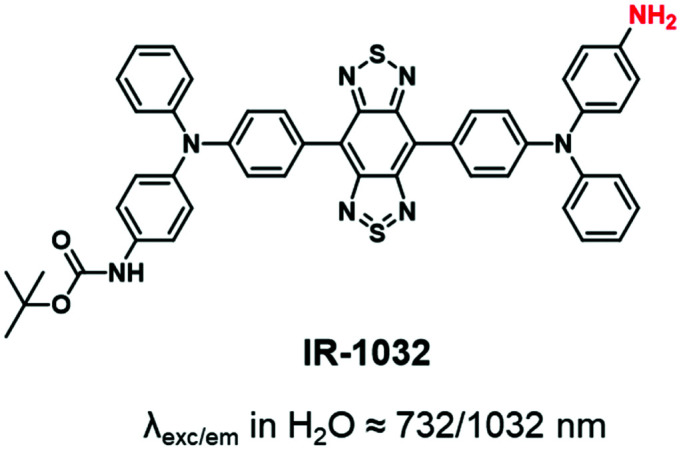
NIR-II dye displaying an amino function for BCP labeling.

Several studies have proved the real interest in the concept of the NIR region considered as the biological transparency window. The transition from the NIR-I to the NIR-II window presents some improvements such as deeper tissue imaging, higher spatial resolution, and higher contrast owing to minimal auto-fluorescence and tissue scattering.^39^ However, the complex multiple synthetic steps and the tedious chromatographic isolation are the main drawbacks for the development of NIR-II dyes.^103^ Consequently, only one NIR-II fluorophore (IR-1032) was involved in the fluorescent labeling of BCPs. IR-1032 bears a free-amino reactive function enabling a covalent linkage with BCP^104^ (Fig. 7).

This section revealed that numerous fluorophores have already been used to label BCPs, covering the visible range and spanning to NIR-I and NIR-II. Additionally, a wide variety of functions have been added to these FMs and used for labeling, either by post-modification of the copolymer (typically using –NH_2_, COOH, N_3_, alkyne, isothiocyanate) or by incorporation into the polymer backbone (typically methacrylate, styrene).

## 2. Synthetic strategies and applications

**Fig. 8:**
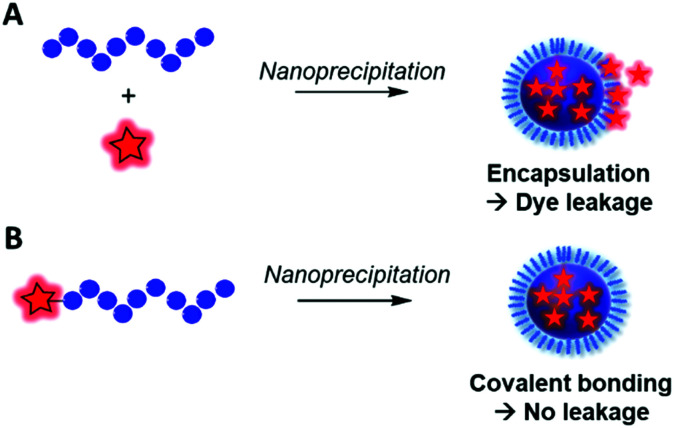
Difference between the fluorescent labeling obtained by (A) encapsulation of a FM that is prone to dye leakage and (B) covalent labeling avoiding the leakage phenomenon.

**Fig. 9:**
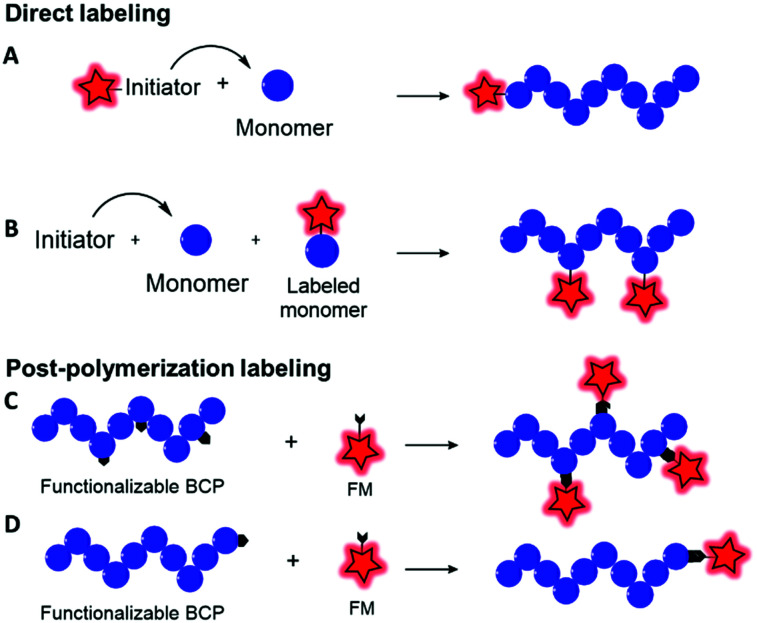
General strategies to incorporate a FM into a block copolymer backbone. Direct labeling by means of a fluorescent initiator (A) or a labeled monomer (B). Post-polymerization labeling using reactive functions on the polymer backbone (C) or at the terminal end (D). The FM is represented as a red star.

Fluorescent labeling of a polymer can be achieved by different methods. As discussed above, the non-covalent encapsulation of a fluorescent marker in a polymer matrix appears to be a simple method and possesses the advantages of keeping the intrinsic properties of the copolymers intact.^105^ However, to avoid the leakage of the dye out of the polymer, especially in biological media, the fluorophore and the polymer should be sufficiently hydrophobic to retain the FM through strong hydrophobic interactions.^26,33,104^ Although this method can be efficient when polymers possessing a highly hydrophobic matrix are combined with fluorophores with enhanced hydrophobicity,^106^ it cannot be applied to all BCPs since the FM will escape (Fig. 8) and thus lead to false conclusions in imaging and potential toxic effects. To prevent this issue, different methods were explored to covalently link the FM to the polymer matrix. Two main methods can be differentiated: direct labeling and post-polymerization labeling (Fig. 9). Importantly, Fig. 9 shows that the different labeling strategies lead to polymers with various densities of FM. “The more, the better” is not necessarily applicable to fluorescent labeling. First, a high density of FM results in a significant chemical modification of the BCP which can affect its physico-chemical properties. Furthermore, unlike the AIE phenomenon where aggregation of the FMs due to their proximity within the polymer matrix leads to fluorescence enhancement, the aggregation of classical FMs (non-AIEgens) lowers the quantum yield and thus decreases the brightness of the labeled BCP, a phenomenon well-known as aggregation caused quenching (ACQ).^32,107^ Overall, the different methods present advantages and drawbacks that are discussed throughout the review.

### 2.1. Direct labeling

Direct labeling consists in incorporating the FM during the polymerization step. Although this method is apparently faster than post-polymerization labeling (*vide infra*), some limitations need to be considered. First, the FMs used in this method are generally not commercially available and hence need to be synthesized accordingly. Then, the FM must be stable in the harsh conditions of polymerization such as high temperatures or the presence of Lewis acids or radicals and should not interfere in the polymerization step. Direct labeling can be proceeded *via* two approaches, namely, using fluorescent initiators or fluorescent monomers.

#### 2.1.1. Direct labeling using fluorescent initiators

**Fig. 10:**
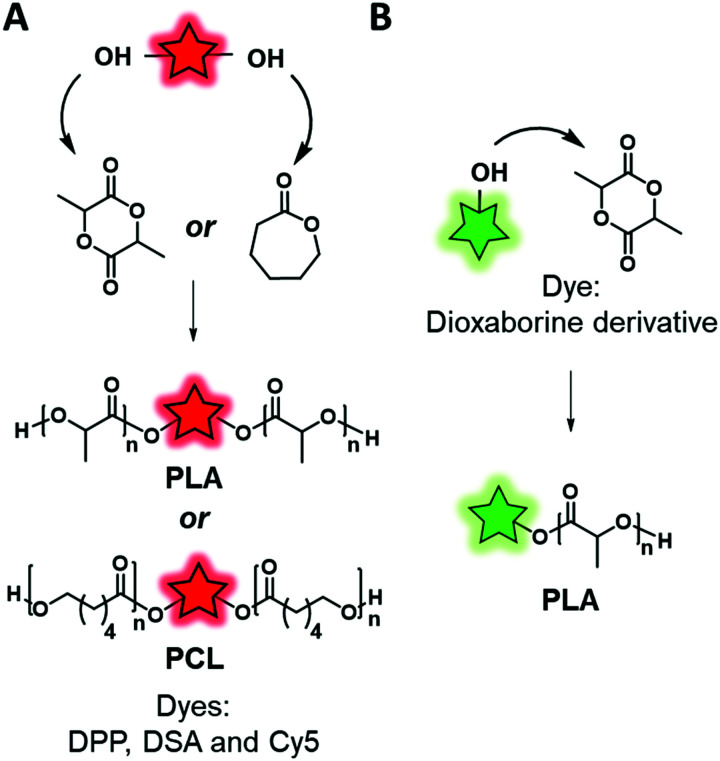
Fluorescent labeling by ROP using a divalent fluorescent initiator (A) or a monovalent one (B).

The “direct labeling” approach consists in using a FM possessing a function that will initiate the polymerization (Fig. 9A). In 2010, Chaney *et al.* used a cyanine 5 bearing two hydroxyl functions (Fig. 1) as the fluorescent initiator of ring-opening polymerization (ROP) of lactide (Fig. 10A) to obtain a fluorescent PLA.^59^ PEG–PLA copolymers were mixed with Cy5–PLA conjugates and co-precipitated in order to form NPs. Once injected into the lymphatic circulation of mice, the biodistribution of NPs was studied by fluorescence *in vivo* imaging, thanks to the far-red emission of Cy5 (see the application in Fig. 17H–J). Although the circulation of NPs did not seem to alter their integrity, after 24 h a low fluorescence signal was recorded in the brain. This signal was compared to the one obtained after the injection of free Cy5. The difference was significant proving the efficacy of the labeling. However, the ester bond between the dye and the copolymer can be hydrolyzed, thus leading to the accumulation of the free Cy5 in the brain.

Similarly, Wang *et al.*^101^ and Zhang *et al.*,^46^ respectively, designed two AIEgen-initiators: diketopyrrolopyrrole (DPP) and an anthracene derivative (DSA-OH) (Fig. 6), both of which bear two hydroxyl groups and can be used to initiate ring-opening polymerization of caprolactone (Fig. 10A). The resulting labeled polycaprolactones (PCL) were then grafted to PEG through an activated carbamate bond ensuring the amphiphilicity of the system. The two research groups used the same material for different applications. Wang *et al.* studied the influence of the polymer matrix on the fluorescence of DPP. Indeed, four copolymers with different molecular weights were synthesized and their photophysical properties were compared to those of the AIEgen itself. The authors showed that high molecular weight copolymers displayed higher brightness due to enhanced aggregation of the AIEgen within the polymer matrix. Zhang *et al.* used this labeled polymer for specific targeting of cancer cells. In addition to the introduction of the DSA AIEgen, they functionalized the PEG–PCL copolymers with folate groups. The resulting NPs showed an ability to target HeLa cells, overexpressing folate receptors, which suggested them as promising cancer cell markers.

FMs based on dioxaborine complexes (Fig. 5B) were widely used by the group of Fraser to develop luminescent responsive polymers.^108–111^ In a recent example, oxygen-sensitive materials were used for wound imaging.^112^ To this endeavor, halogenated (Br or I) dioxaborine-based fluorophores bearing hydroxyl groups were used as initiators in the ROP of lactide (Fig. 10B). After studying the photophysical properties and oxygen sensitivity of the labeled PLA, the iodinated derivative was successfully used as a metal-free alternative fluorescent oxygen-sensitive probe (see the application in Fig. 17A).

Although the efficiency of this approach was demonstrated, it is mainly limited to the ROP that requires a FM bearing hydroxyl groups to initiate the polymerization. It is also important to mention that this approach limits the number of FMs per polymeric chain to one. Interestingly the position of the FM can be controlled. Indeed, if the FM possesses one initiating group (–OH) the polymer chain grows from it and thus the FM is located at one end of the polymer chain. Conversely, when the FM possesses two or more initiating groups, several polymer chains grow from it, forming a polymer labeled at its center (Fig. 10A). Overall, this labeling approach is limited, as it cannot be applied to all the polymerization processes such as radical polymerization. Consequently, direct incorporation of the fluorophore using fluorescent monomers was considered.

#### 2.1.2. Direct labeling using fluorescent monomers

**Fig. 11:**
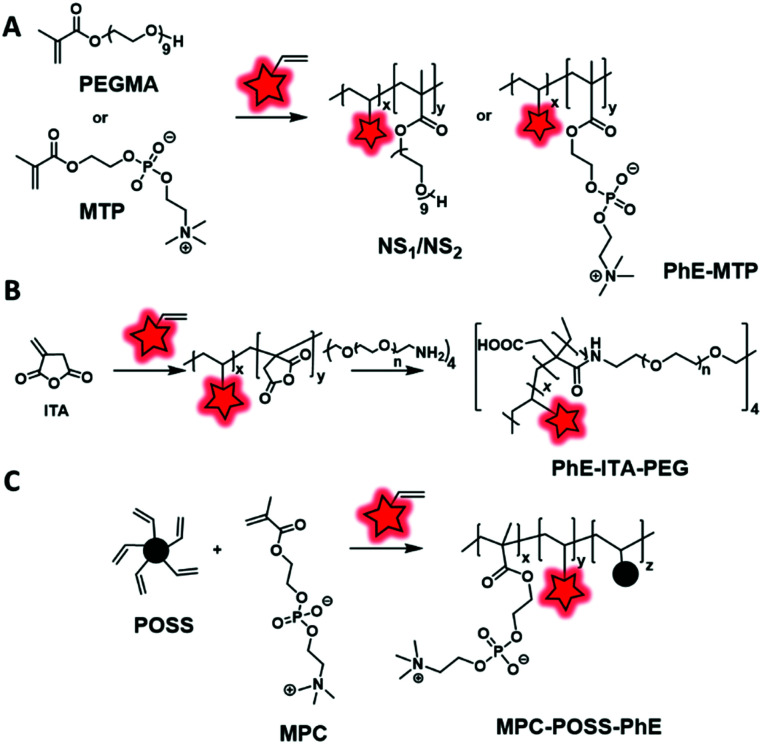
Examples of polymerization using vinyl terminated AIEgens as FMs: (A) PhE-MTP developed by Ma *et al.* and Liu *et al.*, (B) PhE-ITA-PEG described by Ma *et al.* and (C) MPC-POSS-PhE described by Huang *et al.*

The second approach of direct labeling is based on the introduction of fluorescent monomers during the polymerization step (Fig. 9B). For an extensive review of the chemistry of fluorescent monomers as building blocks for labeled polymers, the reader can refer to the work of Schubert and coworkers.^7^ Before its incorporation, the fluorophore is transformed into a monomer through the addition of a polymerizable function (*e.g.* vinyl, acrylate, styrene, allyl), allowing its integration in different polymerization processes. Unlike the use of fluorescent initiators, the dye content can be adjusted by controlling the number of fluorescent monomers incorporated during the polymerization. The first main polymerizable group that can be used is the vinyl moiety. It was used to incorporate AIEgens in BCPs through reversible addition–fragmentation chain-transfer (RAFT) or radical polymerization. Ma *et al.*^45^ and Liu *et al.*^98^ developed two novel AIE polymeric NPs using NSP2E and PhE vinylic dye-monomers, respectively (Fig. 6). These monomers were, respectively, incorporated into PEGMA (poly(ethylene glycol)-(methacrylate)) and MTP (2-methacryloyloxyethyl phosphorylcholine) by RAFT polymerization (Fig. 11A). The resulting polymethacrylates present intense fluorescence (high quantum yields) and have proved their biocompatibility in cell imaging. Another work from Ma *et al.* focused on enhancing the fluorescence brightness of copolymers containing AIEgens.^44^ In this study, the authors developed a cross-linked fluorescent copolymer. Firstly, a vinyl dye-monomer (PhE, Fig. 6) and itaconic anhydride were polymerized by radical polymerization. Subsequently, the obtained polymer was cross-linked by reacting a 4-arm PEG-amine on the anhydride function (Fig. 11B). After formulation, the resulting NPs presented an amphiphilic shell with a cross-linked fluorescent core enhancing the stability of the NPs and ensuring the aggregation of AIEgens, hence increasing their fluorescence. Due to the requirement of their aggregation, most of the AIEgen-based polymeric NPs developed are not stable in dilute solutions. For that reason, Huang *et al.* prepared biocompatible cross-linked copolymers that present an ultra-low critical micellar concentration (CMC) in aqueous solutions.^97^ To achieve this goal, the hydrophobic vinyl dye-monomer PhE (Fig. 6) and the hydrophilic methacrylate MPC (2-methacryloyloxyethyl-phosphorylcholine) were polymerized using a cross-linking agent, POSS (8-vinyl polyoctahedral silsesquioxanes) (Fig. 11C). A series of characterization studies proved their ultra-low CMCs (as low as 7 μg mL^−1^), high water dispersity and photostability.

**Fig. 12:**
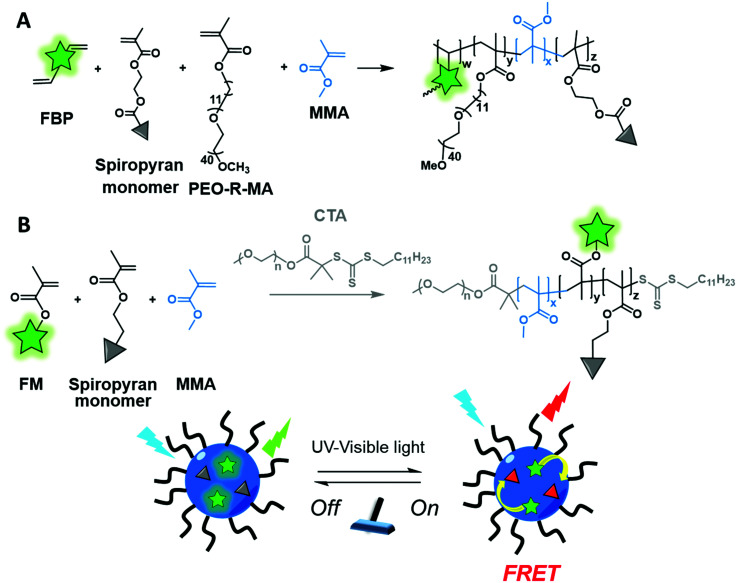
Cross-linked BCP forming photoswitchable NPs with two different pairs of dyes (A) FBP and spiropyran and (B) BODIPY–methacrylate and spiropyran described by the Pinggui group.

Although cross-linking is used to enhance the fluorescence of AIEgen based polymers or to improve the stability of NPs, it can represent another strategy to introduce a fluorophore into a BCP. The Pinggui group developed a novel FRET-mediated photoswitchable system based on a fluorescein-conjugated cross-linking agent.^113^ The authors first synthesized an amphiphilic monomer (PEO-*R*-MA-40) composed of a hydrophilic 40-units PEG chain and a hydrophobic aliphatic chain (C_11_) terminated by a polymerizable methacrylate group. The latter was copolymerized with the fluorescent cross-linker (FBP, Fig. 4B) bearing polymerizable allyl groups and a spiropyran-based photoswitcher (SPMA) bearing a methacrylate group (Fig. 12A). This design allowed core–shell NPs emitting in the green to be obtained due to the fluorescein cross-linker. Upon irradiation the spiropyran opens up and leads to a red emitting merocyanine that acts as a FRET acceptor. This photoswitchable system is promising for biological labeling due to its improved properties such as biocompatibility, tunable FRET efficiency and high photoreversibility. Similarly, the same group also used methacrylate modification for BCP labeling.^48^ The authors developed both fluorophore and photochromic methacrylate monomers: BODIPY–MA (Fig. 5A) and spiropyran–MA, respectively, which were incorporated into the PEG–PMMA (poly(ethylene glycol–methyl methacrylate)) backbone (Fig. 12B). The resulting NPs presented similar photoswitchable properties to those described above due to the FRET phenomenon.

**Fig. 13:**
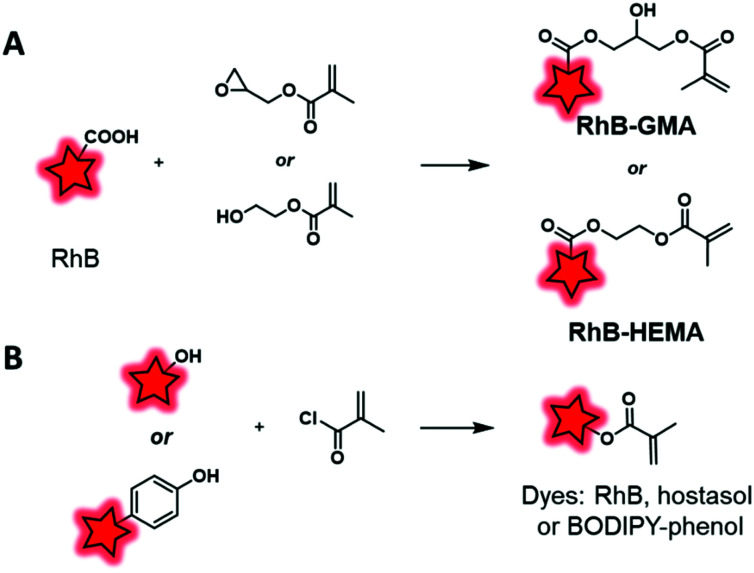
Transformation of fluorophores into dye-methacrylate monomers by esterification with GMA or HEMA (A) or by esterification of methacryloyl chloride (B).

The second main polymerization moiety that is linked to FM is methacrylate. A common method consists in esterifying the hydroxyl end group of HEMA (2-hydroxyethyl methacrylate) or the epoxy function of GMA (glycidyl methacrylate) monomers using the carboxylic acid of rhodamine B (Fig. 4A). Then, these labeled monomers were copolymerized with MMA (methyl methacrylate) through free-radical emulsion polymerization (FREP) or surface-grafting polymerization (Fig. 13A).^114^ The corresponding NPs were used as new fluorescent trackers for cells in *in vitro*^115^ and *in vivo*^116^ studies. Using the dye-monomer RhB–HEMA (Fig. 13A), Sitia *et al.* developed two types of biodegradable BCPs: PLA and PCL.^117^ In order to polymerize with the dye-monomer, macromonomers (PLA, PCL and PEG) were modified to display methacrylate functions. Practically, macromonomers were synthesized through ROP using HEMA as the initiator. Then, polymeric NPs were produced by FREP of three monomers, RhB–HEMA, PEG–HEMA, PLA–HEMA or PCL–HEMA. The NPs were injected in mice in order to compare their behavior and biodistribution *in vivo*. Thanks to the rhodamine FM, several features were studied including stability, bloodstream permanence, biodistribution, and tumor penetration. In conclusion, PCL-based NPs were revealed as a highly promising material for the development of drug nanocarriers (see the application in Fig. 17B–G). In parallel, Nicolas *et al.* showed the possibility to fluorescently tag a copolymer by incorporation of RhB or hostasol labeled methacrylate monomers (Fig. 3 and 4A, respectively).^73^ Fluorophores were designed to bear hydroxyl groups that were esterified as methacrylates using methacryloyl chloride (Fig. 13B). The labeled copolymers were obtained by copolymerization with PEGMA through living radical polymerization using macroinitiators based on proteins including bovine serum albumin (BSA) and lysozyme. Bioconjugation with proteins is an original approach, which is complementary to PEGylation. Similarly, Charleux and co-workers developed a methacrylate–BODIPY (Fig. 5A) by the esterification of BODIPY–phenol with methacryloyl chloride (Fig. 13B).^87^ The monomer was copolymerized with poly(ethylene oxide)methyl ether acrylate and acrylic acid through mini-emulsion RAFT polymerization to obtain pH-responsive polymeric NPs. Using the same methacrylate-BODIPY, green fluorescent polymer chains (GFPC) were synthesized which presented interesting features such as non-toxicity, biocompatibility, water-solubility and stability in complex media.^88^ This polymer was successfully used to label bacteria in multicolor bioimaging and for their detection by flow cytometry.

**Fig. 14:**
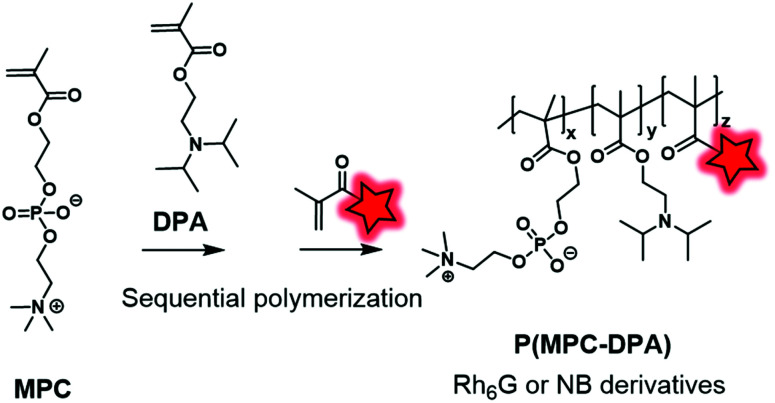
P(MPC–DPA) BCP fluorescently labeled by Nile Blue and rhodamine-6G derivatives described by the Madsen group.

As an alternative to the esterification reaction, methacrylic monomers were linked to FMs *via* amide coupling or by the formation of carbamate and urea function. Recently, Madsen *et al.* synthesized Nile Blue monomers such as Nile Blue methacrylamide (NBM) and Nile Blue 2-(methacryloyloxy)ethyl urea (NBU) (Fig. 3).^74^ Unlike previous examples, Nile Blue-based monomers were added at the end of the copolymerization between MPC (2-methacryloyloxyethyl-phosphorylcholine) and DPA (2-(diisopropylamino)ethyl methacrylate) monomers (Fig. 14). Indeed, kinetic studies of the polymerization using Nile Blue precursors revealed a problem of retardation due to the stabilization of Nile-Blue radicals induced by intramolecular reactions. To circumvent this issue, the dye-monomers were incorporated after 80% conversion of monomers. Thanks to the shifted p*K*_a_ of the Nile Blue monomers compared to its parent compound, the resulting copolymers displayed pH sensitivity in the physiological range. Thanks to their fluorescent pH sensing properties, these BCPs can be utilized to detect acidic regions in tumors. Using the same strategy, the authors also developed several rhodamine-6G derivatives (Fig. 4A) that were modified into methacrylate monomers by amidation of carboxylic acid.^118^ During the copolymerization no retardation was observed and thus the fluorophores were statistically located in the polymer backbone. The resulting biocompatible P(MPC–DPA) BCP (Fig. 14) could be used for various intracellular delivery experiments.

**Fig. 15:**
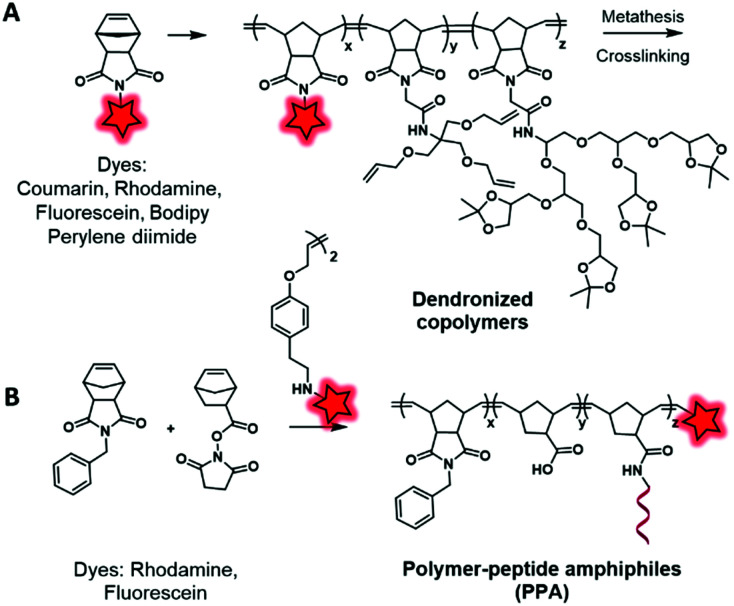
Norbonenyl-based BCPs described by Zimmerman (A) and Gianneschi (B) groups.

In another approach, labeled monomers bearing the norbonenyl moiety can be involved in ring-opening metathesis polymerization (ROMP) using Grubbs’ catalyst. Li *et al.*^119^ developed cross-linked copolymers in order to demonstrate the influence of the polymer matrix on the stability of dyes. To this endeavor, the authors incorporated fluorescent norbonenyl monomers, including coumarin, fluorescein, BODIPY, perylene diimide and rhodamine, into cross-linked dendronized polyol by ROMP (Fig. 15A). As expected, the use of polyglycerol dendrimers enhances the aqueous solubility of the resulting polymeric NPs which are promising for cell imaging. Using this approach, Gianneschi and his team^120^ prepared two dye-ended BCPs using symmetric olefins of dye dimers that were incorporated at the end of the polymerization process. These BCPs were modified with peptide sequences in order to generate enzyme-responsive NPs and to explore the influence of peptide-grafting on the tumor accumulation (Fig. 15B). The green and red NPs were co-injected in mice and, using FRET *in vivo* imaging, the authors were able to track the accumulation of both NPs in tumors as well as their degradation within the latter. In the tumor environment, the NPs underwent enzyme cleavage and rearranged into new structures containing both fluorophores, allowing a new FRET-active signal. Further control experiments were done with NPs without peptide sequences and no FRET was observed, which demonstrated the influence of peptide-grafting.

#### 2.1.3. Direct fluorescent labeling using multicomponent reactions

**Fig. 16:**
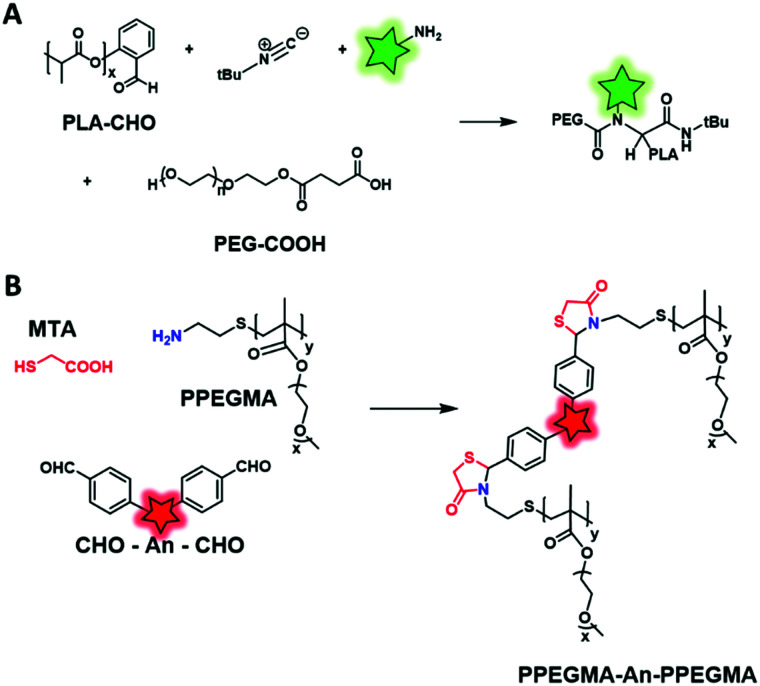
Direct fluorescent labeling of a BCP using Ugi (A) and MALI (B) multicomponent reactions.

Besides the use of fluorescent initiators and monomers, other approaches have been used to perform direct fluorescent labeling of BCPs. Von der Weid *et al.* proposed an original approach to synthesize a PEG–PLA BCP through a Ugi multicomponent reaction (MCR)^121^ involving four components, namely, a ketone or an aldehyde, an amine, an isocyanide and a carboxylic acid, to form a bis-amide (Fig. 16A). The aldehyde function was introduced by the salicylaldehyde that served as the initiator in the ROP of lactide. PEG was functionalized with succinic anhydride forming a carboxylic acid end. To these components were added fluorescein-NH_2_ (commercially available, Fig. 4B) and *tert*-butyl isocyanide enabling the Ugi reaction. This reaction allowed the preparation of the PEG–PLA BCP with a fluorescent label connecting both blocks.

**Fig. 17:**
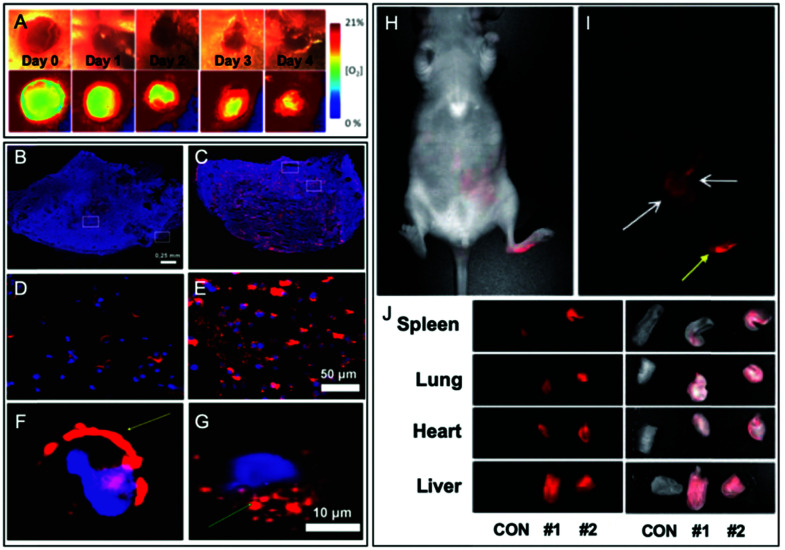
Examples of fluorescent BCPs obtained by direct labeling used in bioimaging applications. (A) Ratiometric imaging of wound oxygenation and healing over time using fluorescently labeled PLA. Adapted with permission from ref. 112. Copyright (2016) American Chemical Society. (B–G) Difference of uptake in the tumor section (B and C) and cancer cells (D–G) between NPs composed of fluorescently labeled BCPs based on PLA (B, D and F) and PCL (C, E and G). Adapted with permission from ref. 117. Copyright (2016) American Chemical Society. (H–I) *In vivo* imaging (24 h post-injection) of fluorescently labeled PLA based NPs in nude mice and their biodistribution in representative organs (J). Yellow and white arrows in (I), respectively, represent the injection point and the distribution of NPs. In (J), CON represents a control nude mouse and #1 and #2 represent the injected mice. Adapted with permission from ref. 59. Copyright (2010) Sage Publishing.

Similarly, Wan *et al.* described the synthesis of PPEGMA-An-PPEGMA through a mercaptoacetic acid locking imine (MALI) multicomponent reaction.^99^ PEGMA monomer was modified into an amino-terminated copolymer by chain-transfer free radical polymerization using cysteamine hydrochloride as the chain transfer agent (CTA). Then, an AIEgen was functionalized with two benzaldehydes (DSA–CHO, Fig. 6), allowing the formation of thiazolidin-4-one with PEGMA and mercaptoacetic acid (MTA) (Fig. 16B). Finally, the resulting NPs were tested in cell imaging and showed excellent biocompatibility. This original strategy presents several advantages such as the mild conditions of labeling (room temperature, catalyst-free) and the possibility to conjugate amino-containing biomacromolecules.

The MCR approach presents an attractive alternative for the synthesis of fluorescent BCPs since three or more components with various properties can be involved in one reaction. Moreover, it showed appealing features such as the atom economy and high efficiency using mild conditions (room temperature and without catalyst) which are favorable to the introduction and the integrity of the FM. However, this rather new approach requires prior preparation of the functional components, which appears quite time consuming.

Overall, the approach consisting in the direct introduction of a fluorescent monomer during the polymerization step seems appealing. Indeed, this approach allows control over the number of FMs per polymer chain which, unlike the “fluorescent initiator” approach, can be more than one. However, special attention should be paid to the possible effect of the labeled monomer on the polymerization reaction. Indeed, this section reports some cases wherein the labeled monomer displayed different polymerization kinetics compared to the parent monomer and can thus delay or inhibit the polymerization. Moreover, the present section reveals that, unlike the “fluorescent initiator” approach, the “fluorescent monomer” one is exclusively reserved for radical polymerization. In fact, to our knowledge, fluorescent monomers that can be used in ROP have not been reported yet.^7^ This observation might be due to the relative instability of monomers like glycolide, lactide and caprolactone that can hardly tolerate modifications. Finally, the chemical stability of the FM must be considered since the harsh polymerization conditions could destroy it or alter its properties.

### 2.2. Post-polymerization labeling

The second main approach to obtain a fluorescently labeled BCP consists in covalently grafting a FM once the polymer is obtained. The labeling is generally enabled through functions borne by monomers (typically COOH from methacrylates) or by initiators, thus placing the fluorophore along the polymer backbone or at the terminal position, respectively. Various coupling reactions have been used including amide coupling, isothiocyanate coupling, click reaction or Mitsunobu reaction. This approach ensures a better integrity of the fluorophore as the fluorescent labeling of BCP is generally achieved in milder conditions than those required for their polymerization.

#### 2.2.1. Post-labeling on the polymeric backbone

**Fig. 18:**
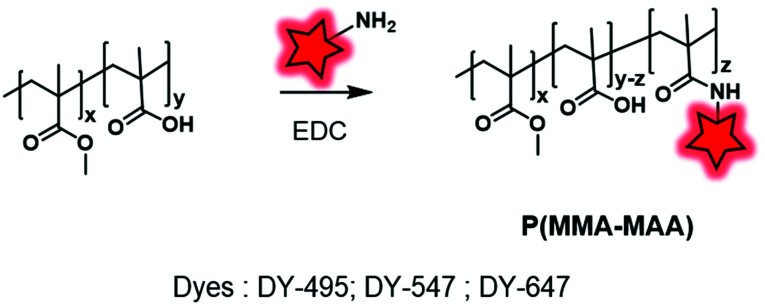
Post-labeling of P(MMA–MAA) with DY-469, DY-547 and DY-647 using the backbone carboxylate functions.

Among post-polymerization labeling reactions, those involving the carboxylate of the polymethacrylate backbone appear to be the simplest. Schubert and coworkers labeled differently sized NPs to study their relative cellular uptake.^57^ To this endeavor, the carboxylate functions of the poly(methyl methacrylate–methacrylic acid) P(MMA–MAA) BCP were reacted with three different amino-dyes (DY-495, DY-547 and DY-647, Fig. 1 and 4B) to obtain a color code in bioimaging (Fig. 18).

**Fig. 19:**
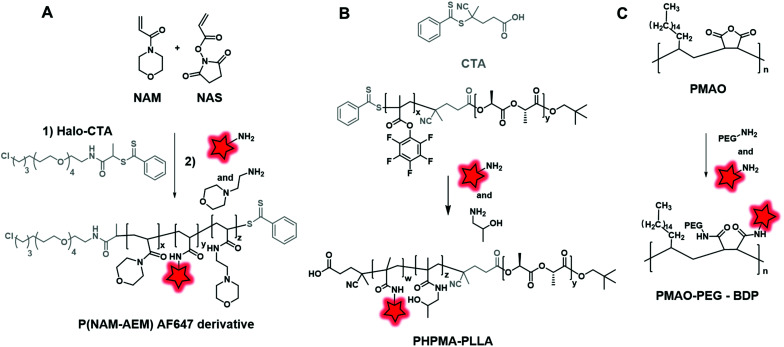
Synthesis and fluorescent labeling of BCPs P(NAM–AEM) (A), PHMA–PLLA (B) and PMAO–PEG (C) using amine reactive functions: activated NHS, pentafluorophenol esters and anhydride functions, respectively.

Amide coupling can be simplified by the use of active esters such as the NHS ester incorporated in the polymer backbone and it readily reacts with amine, thus avoiding the use of coupling agents. Different studies demonstrated the efficiency of this method.^51,53^ Favier and coworkers described a method to synthesize a fluorescent polymer to specifically and covalently label membrane proteins.^58^ First, a chain transfer agent (CTA) containing a HaloTag ligand was developed and involved in the RAFT copolymerization of *N*-acryloylmorpholine (NAM) and *N*-acryloxysuccinimide (NAS) to obtain a HaloTag-terminated poly(NAM–NAS) polymer. Then, the activated NHS esters, distributed along the polymer backbone, were reacted with amino-AF647 (Fig. 1) and capped with aminoethylmorpholine (AEM) (Fig. 19A). Finally, the far-red emitting BCP was used to label Halo-tagged surface proteins with great potential in super-resolution imaging. Interestingly, Zentel and coworkers developed an amino-reactive diblock copolymer, P(PFMA–PLLA) (poly(pentafluorophenyl methacrylate–l-lactic acid)). First, the authors coupled PLA to a CTA that was then used to promote the RAFT polymerization of amino-reactive pentafluorophenyl methacrylate (PFMA).^122^ Oregon Green 488 cadaverine (Fig. 4B) served to fluorescently label the polymer and the residual PFMA was capped with hydroxypropyl amine leading to the biocompatible fluorescent polymer PHPMA–PLLA (Fig. 19B). In our recent work, the amino reactive-anhydride functions of poly(maleic anhydride-*alt*-1-octadecene) (PMAO) were reacted with an amino-PEG and a bulky amino-modified BODIPY (Fig. 5A) in a one-pot reaction, resulting in a fluorescent amphiphilic BCP (Fig. 19C). In aqueous media, the latter folds into monomolecular NPs which possessed a small size (10–14 nm) and impressive brightness due to the bulkiness of the BODIPY that prevented the ACQ phenomenon.^89^

**Fig. 20:**
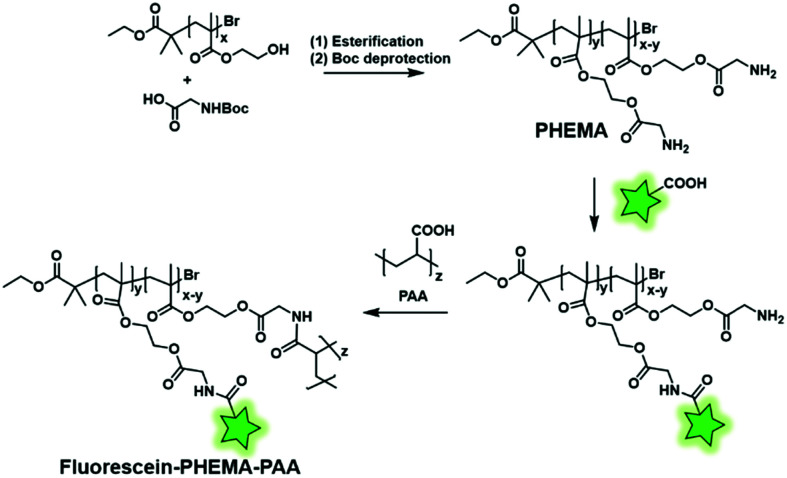
Fluorescent labeling of the PHEMA–PAA BCP by the introduction of amino functions.

Reciprocally, the fluorescent labeling can be performed between a FM bearing a carboxyl function and a BCP bearing amino groups. Min and coworkers first synthesized a poly(hydroxy(ethyl)methacrylate) (PHEMA) copolymer by RAFT polymerization before esterifying the hydroxyl groups by *N*-Boc glycine.^123^ After Boc deprotection, the polymer backbone possessed several free amino functions enabling the amide coupling with a carboxyfluorescein using a coupling agent (Fig. 4B). The remaining free amino functions of the resulting fluorescent copolymers were then coupled to NHS-activated poly(acrylic acid) (PAA) in order to balance the hydrophilicity of the system (Fig. 20). Finally, the polymer was formulated to obtain fluorescent NPs that were labeled with aptamers, enabling their use in *in vitro* imaging of cancer cells and long-lived *in vivo* imaging of tumors.

**Fig. 21:**
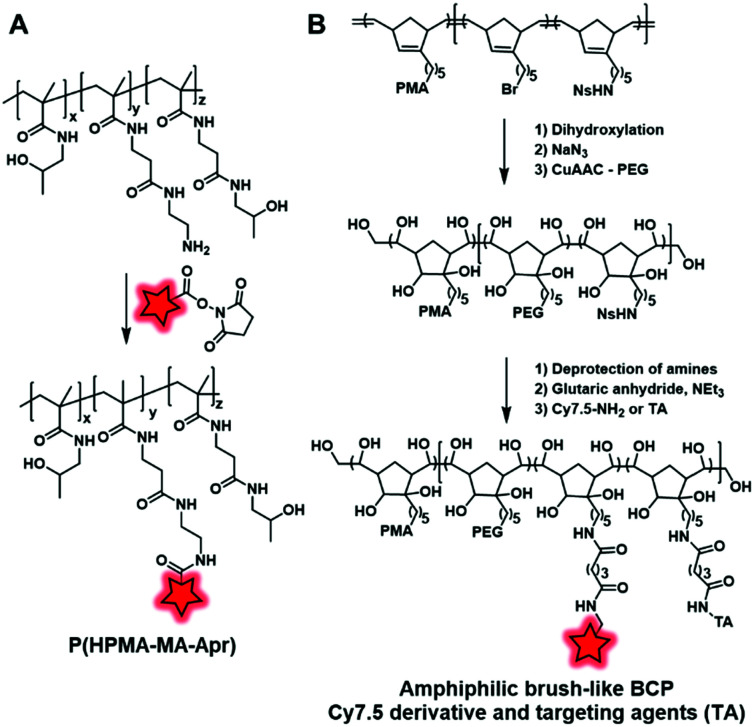
(A) Polymethacrylate BCP developed by Weiss *et al.* in order to coat PGAS NPs; (B) PMA norbornadiene backbone functionalized with PEG, Cy7.5 and targeting agents (TA).

Weiss *et al.* developed a polymethacrylate BCP (P(HPMA–MA–Apr)) (Fig. 21A) displaying free amino groups which were coupled with the amino-reactive fluorophore DY-676–NHS (Fig. 2).^65^ Then, the fluorescent BCP was used to coat PGAS-based NPs (stearic acid-modified poly(glycerol adipate)) in order to decrease the surface charge of NPs and to show that it did not influence their accumulation in the tumor.

Miki *et al.* also used amidation to develop fluorescent NPs based on an amphiphilic brush-like copolymer.^124^ The polymeric backbone obtained by metathesis polymerization is composed of three norbornadiene monomers bearing, respectively, a bromide, a protected amine function (nitrobenzenesulfonyl amine NHNs), and a poly(methacrylate) (PMA) as hydrophobic chains. Then, the alkene groups were dihydroxylated, followed by the addition of hydrophilic PEG chains through a click reaction enabled by the substitution of bromide into azide. Finally, the amine functions were deprotected and reacted with glutaric anhydride to form carboxylic acid functions, which were subsequently coupled to Cy7.5–NH_2_ (Fig. 1) and different targeting agents (folic acid, RGD and galactosamine) (Fig. 21B). Once injected in mice, the resulting NPs accumulate in targeted tumors with a significant difference in efficiency depending on the targeting agents (see the application in Fig. 34A–D). Although this approach led to new brush-like nanomaterials, it is noteworthy that it required a substantial number of synthetic steps, which limits its broad applications.

**Fig. 22:**
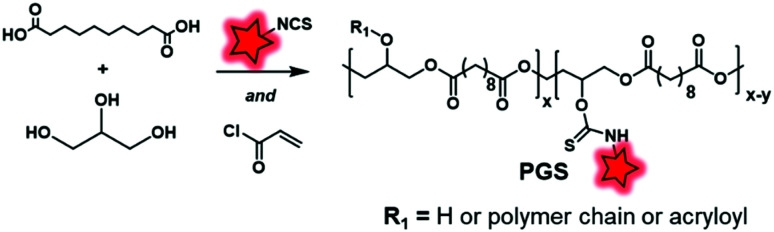
Synthesis and fluorescent labeling of the PGS BCP.

Another labeling approach is described by Xu *et al.*, which is based on the well-known isothiocyanate chemistry.^125^ A poly(glycerol-sebacate) acrylate (PGS) was synthesized by the condensation of sebacic acid and glycerol (Fig. 22). The fluorescent labeling was enabled by hydroxyl free functions along the polymer backbone, which reacted with isothiocyanate rhodamine (RITC, Fig. 4A), forming a thiocarbamate link. After demonstrating the advantage of FM-conjugation over FM-encapsulation by cellular imaging and whole animal *in vivo* imaging (see the application in Fig. 34E–H), the authors used the fluorescent NPs to efficiently track stem cells both *in vitro* and *in vivo*.

**Fig. 23:**
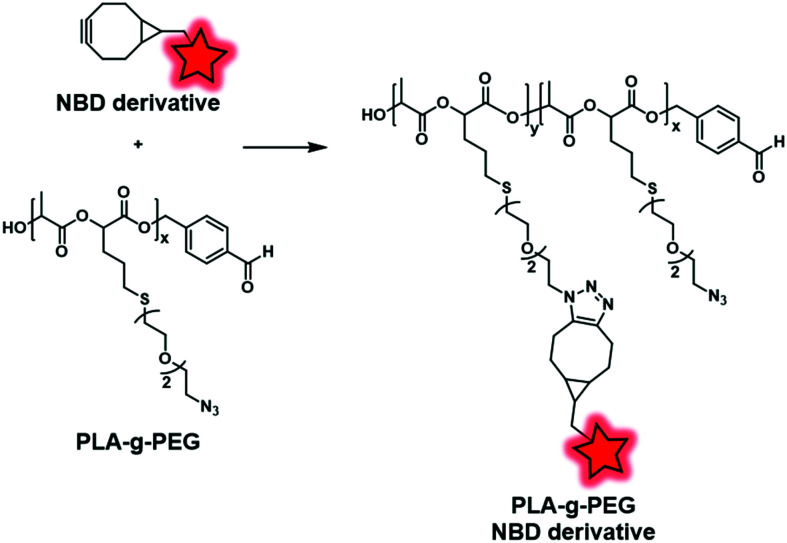
PLA-*g*-PEG BCP fluorescently labeled with the NBD dye *via* SPAAC reaction.

More recently, the interest in click chemistry has grown due to its environment-friendly approach, fast process and mild conditions.^126^ Among bioorthogonal click reactions, Huisgen cycloaddition involving the reaction of an alkyne with an azide and leading to a triazole moiety is the most representative example. This reaction is performed following two different approaches: copper-catalyzed^127^ or strain-promoted^128^ alkyne-azide cycloaddition (CuAAC and SPAAC, respectively). The difference lies in the activation of the reaction. Whereas copper(i) ions are required for CuAAC, SPAAC must involve a constrained alkyne, thus avoiding the use of any catalyst. Consequently, SPAAC offers milder conditions and thus may be used for unstable fluorophores or polymers. Weck and coworkers adopted this reaction in order to label PLA-*graft*-PEG copolymers (Fig. 23).^70^ First, the authors used a functionalized lactide bearing an azido terminated PEG. The latter was involved in ROP to obtain the clickable PLA-*g*-PEG. Subsequently, a NBD dye bearing a bicyclononyne (Fig. 3) was successfully embedded into the copolymer *via* SPAAC reaction.

**Fig. 24:**
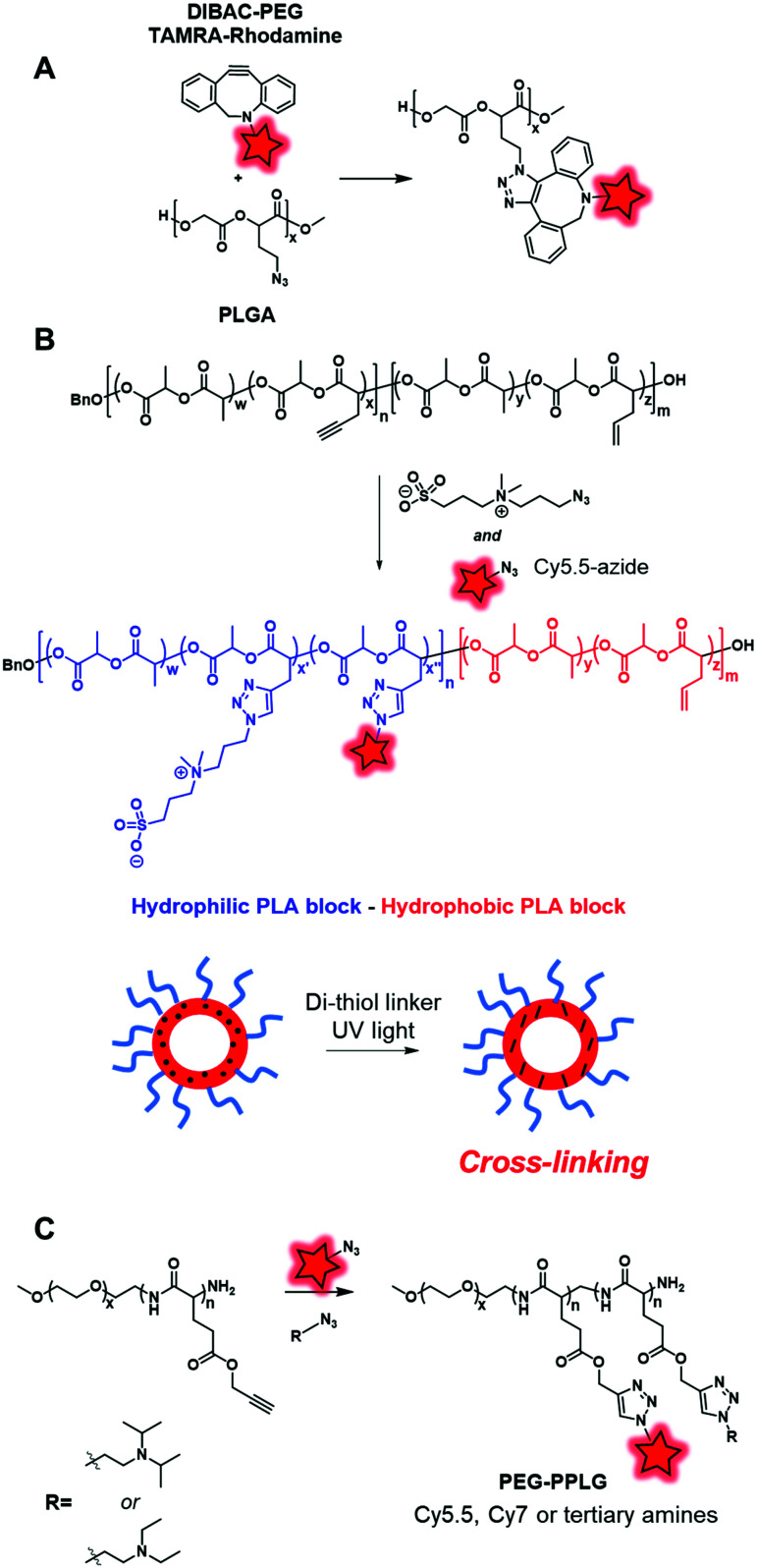
(A) Fluorescent labeling along the PLGA backbone with a rhodamine derivative under SPAAC conditions. (B) Acetylenyl-allyl-PLA was labeled with Cy5.5 and hydrophilic zwitterion under CuAAC conditions. (C) Alkynyl-PEG–PPLG was reacted with Cy5.5, Cy7 and tertiary amines under CuAAC conditions.

In the same way, Almutairi and coworkers synthesized a clickable PLGA (poly(lactic and glycolic acids)) copolymer using an azido glycolide.^129^ Due to this functional moiety, the copolymer was labeled with both the DIBAC–PEG–TAMRA fluorophore (Fig. 4A) and bicyclononyne-folate *via* SPAAC reaction (Fig. 24A). The developed BCP presented the ability to degrade through hydrolysis combined with the ability to be functionalized, making it a promising tool in drug delivery and targeting applications.

Cheng and coworkers also used a clickable polyester to develop a cross-linked amphiphilic PLA block copolymer forming biodegradable nanocapsules.^47^ The PLA diblock copolymer was obtained by two sequential ROP reactions. First, a ROP reaction involving lactide and acetylenyl-functionalized lactide monomers was initiated by benzyl alcohol. Then, the resulting polymer was copolymerized with lactide and allyl-functionalized lactide constituting the second block of the polymer (Fig. 24B). The alkyne groups in the first block polymer chain were successfully reacted with Cy5.5-azide (Fig. 1) and azide-functionalized sulfobetaine zwitterion, resulting in a hydrophilic block. This copolymer was used to prepare nanocapsules by mini-emulsion cross-linking between allyl groups and the di-thiol crosslinker through a UV-induced thiol–ene click reaction. The resulting nanocapsules were studied in *in vivo* imaging where the fluorescence signal could only be detected in tumor sites 24 h post-injection. This successful accumulation showed the promising use of zwitterionic polymer-based materials in cancer imaging. Hammond and coworkers designed PEG–PPLG (propargyl-l-glutamate) block copolymers as pH-sensitive drug carriers.^56^ The hydrophobic part that contained clickable alkyne groups was successfully labeled by Cy5.5-azide or Cy7-azide (Fig. 1) and functionalized with tertiary amines under CuAAC conditions (Fig. 24C). The resulting self-assemblies were injected in mice where they accumulated in tumors. FRET monitoring was established to study the disassembly of NPs at the low pH of the tumor environment. Furthermore, Doxorubicin, an anti-cancer drug, was encapsulated into these fluorescent NPs. With the degradation of NPs at low pH, Doxorubicin was delivered and the tumor growth was significantly reduced.

These examples illustrate the power of the fluorescent labeling of BCPs as it offers a large number of possibilities, namely: the tracking of NPs, the monitoring of their integrity and the evaluation of the drug delivery efficiency.

#### 2.2.2. Post-labeling at the terminal end of the polymer

**Fig. 25:**
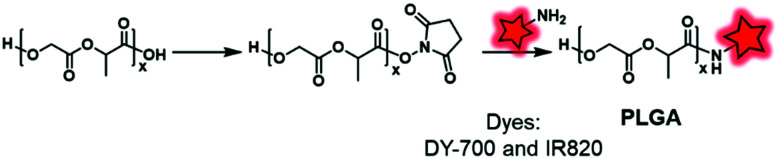
Fluorescent labeling of the end chain of PLGA through NHS active ester.

Another approach to post-labeling consists in using the terminal functions borne by the BCP. The strategies to be applied are determined by the type of polymers as, for instance, polyesters and polyacrylates do not involve the same chemistry. In the case of polyesters prepared from glycolide, lactide or lactones as the monomer, the terminal end bears a hydroxyl function, unlike the polyacrylate backbone that generally does not display any reactive function. The other terminal end, where the polymerization was initiated, can bear a carboxylic function if the polymerization was initiated by water or any other functionality brought by a molecule bearing an amine or a hydroxyl function (to initiate the ROP) and a side function. As explained above, both carboxylate and hydroxyl functions can directly serve to introduce a FM. In complementary works from Fattal^49^ and Arruebo^50^ the terminal carboxylic acids of PLGA copolymers were activated with NHS esters before reacting with amino-fluorophores, DY-700 and IR-820, respectively (Fig. 1 and 2). Biocompatibility, biodistribution and persistence were successfully studied by *in vitro* and *in vivo* fluorescent tracking, showing that these materials can be further used for localized drug release and bioimaging (Fig. 25).

**Fig. 26:**
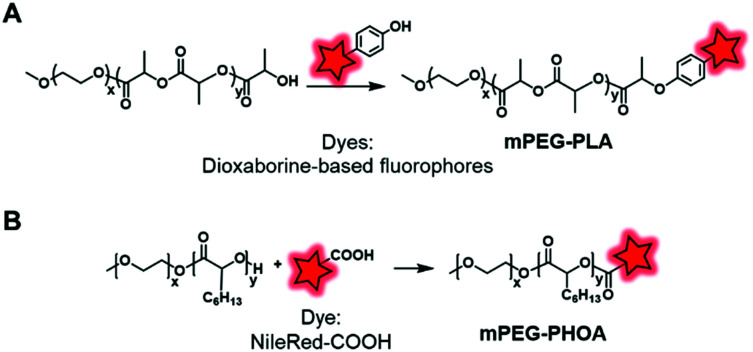
Use of the Mitsunobu reaction to label mPEG–PLA (A) and mPEG–PHOA (B) at their terminal hydroxyl ends.

In an original approach, Kerr *et al.* synthesized a library of fluorescent/phosphorescent dioxaborine-based fluorophores (Fig. 5B) bearing a phenol group which enables post-polymerization labeling *via* the Mitsunobu reaction on the terminal hydroxyl of PEG–PLA (Fig. 26A).^91^ Thanks to the structural diversity of fluorophores, the resulting copolymers were employed to obtain NPs of various emission colors. The long phosphorescence lifetimes and the distinct fluorescence and phosphorescence signals showed the potential of these materials to be used in hypersensitive oxygen sensing. The Mitsunobu reaction was also employed by Möller and coworkers to label a methoxy(PEG)–PHOA (poly(hydroxyoctanoic acid)) copolymer (Fig. 26B).^72^ This BCP bears a free hydroxyl group at its terminal end which was substituted by Nile Red-COOH (Fig. 3). The successful results on cells indicate its great potential for use in biodegradable drug delivery systems.

##### 2.2.2.1. Post-labeling by modification of the terminal end

**Fig. 27:**
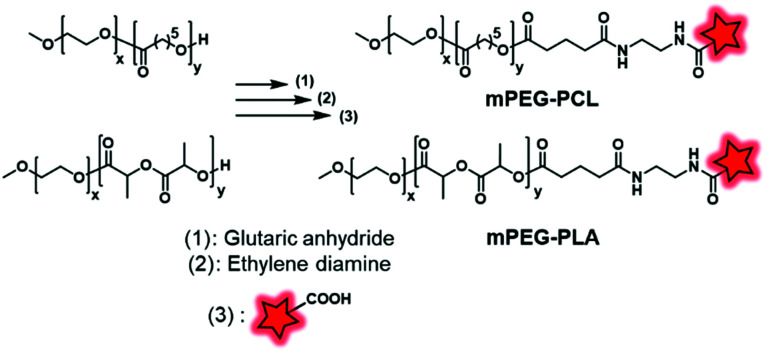
mPEG–PCL and mPEG–PLA fluorescently labeled with Cy5.5 and Cy5 described by Shen and coworkers.

Although the labeling of the terminal end is possible, it sometimes requires slight modifications in order to fit with the available chemical function of the FM. For instance, Shen and coworkers, after synthesizing PEG–PCL and PEG–PLA copolymers, modified the terminal hydroxyl functions to obtain carboxylates *via* a reaction with glutaric anhydride (Fig. 27).^60^ After NHS activation, the BCPs were then amidified by ethylenediamine enabling a coupling with a pair of FRET fluorophores: Cy5–NHS and Cy5.5–NHS (Fig. 1). The *in vivo* behavior of the NPs was studied by monitoring the FRET signal giving access to information on their disassembly and blood clearance to finally improve their therapeutic efficacy (see the application in Fig. 34I).

**Fig. 28:**
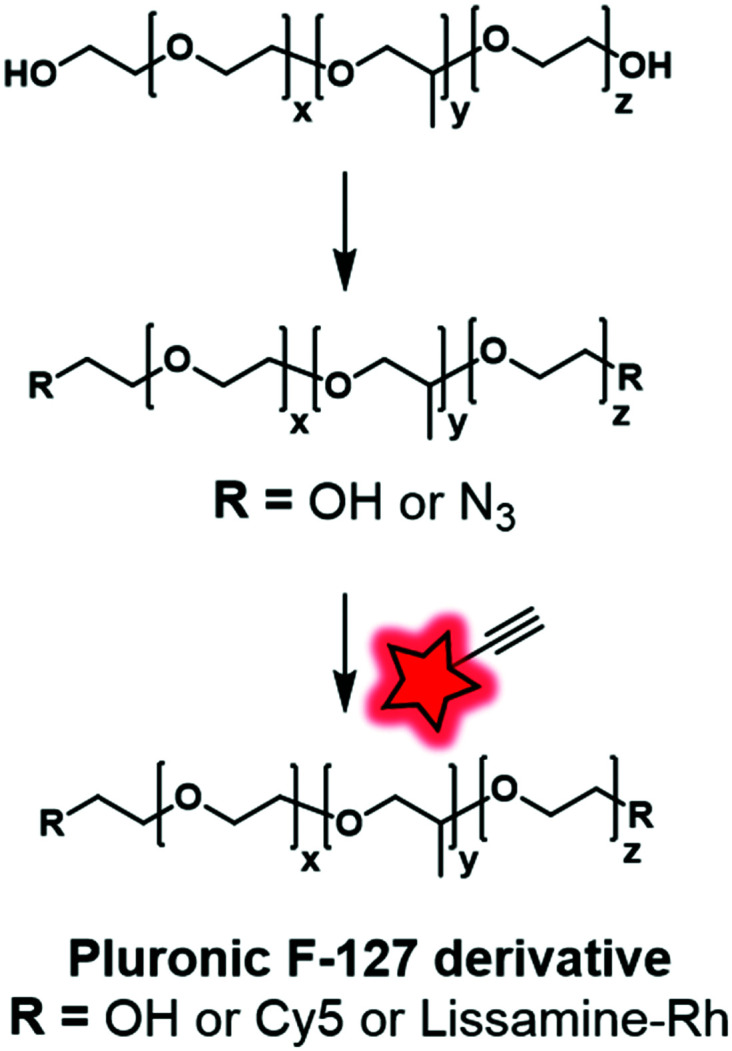
Fluorescent labeling of Pluronic F-127 with Cy5 and Lissamine-Rh by click chemistry described by Reisch and coworkers.

In our recent work, Pluronic F-127 was fluorescently labeled.^55^ Firstly, the hydroxyl functional groups were mesylated before being replaced by azide functions. The modified Pluronic polymer was then reacted under CuAAC conditions with lissamine (a sulfonated rhodamine) or Cy5 (Fig. 1 and 4A) bearing an alkyne group (Fig. 28). The Cy5-conjugate was then used to study the adsorption and stability of the shell at the surface of dye-loaded PMMA–MAA based nanoparticles for further application in live tracking in the mouse brain.

##### 2.2.2.2. Post-labeling by modification of the initiator

**Fig. 29:**
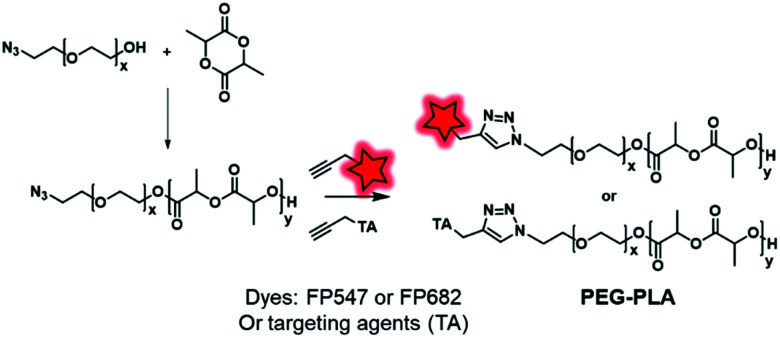
Copper-catalyzed click chemistry on PEG–PLA with fluorophores or targeting agents (TA).

The labeling of the terminal end of polyesters can also be performed using an initiator. For instance, Couvreur and coworkers introduced a clickable azide moiety at the terminal end of a PEG–OH that initiated the ROP of a lactide to obtain the PLA–PEG–N_3_ BCP.^130^ The diblock copolymers were successfully tagged under CuAAC conditions with fluorescent probes (FP547, FP682, commercially available FMs) and specific ligands including biotin, folic acid or anisamide (Fig. 29), enabling the targeting of cancer cells.

**Fig. 30:**
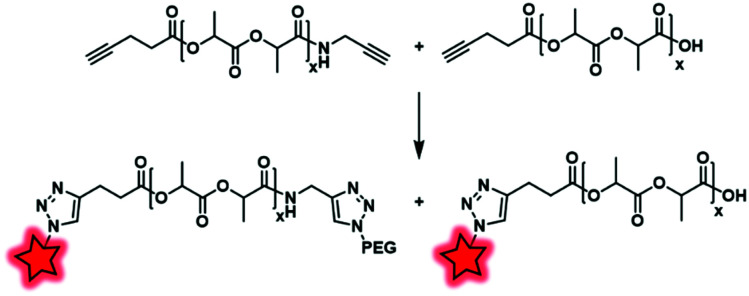
Alkynyl-PLA reacted with Azide-Fluor 545 and methoxy(PEG)-N_3_ under CuAAC conditions.

Based on a similar principle, Scala *et al.* introduced an alkyne function at the terminal end of PLA.^80^ The transesterification of PLA with propargyl alcohol is widely used; however, this method leads to an impressive cleavage of the PLA chain. To circumvent this issue, the authors showed that alkyne-ended PLA can be obtained by reacting propargylamine on PLA in a solvent-free reaction, followed by the esterification of free hydroxyl-end groups with pentynoic anhydride (Fig. 30). The resulting copolymers are randomly terminated by one or two alkyne functions, allowing their functionalization by “clickable” moieties such as Azide-Fluor 545 (Fig. 4A) and methoxy(PEG)-N_3_. This approach avoids the risks of cleavage and thus provides alkynyl-PLA with expected molecular weights.

##### 2.2.2.3. Post-labeling by modification of the CTA

**Fig. 31:**
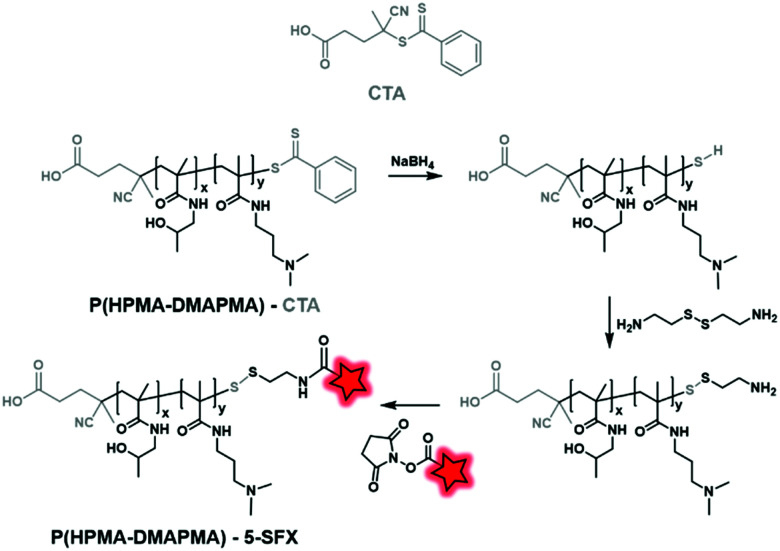
Post-modification of the CTA enabling the fluorescent labeling of 5-SFX.

Unlike polyesters, the polyacrylate/polyacrylamide BCP does not necessarily possess targetable functionalizable sites at their ends. However, those obtained by RAFT polymerization display reactive functions at their end due to the presence of the chain transfer agent (CTA), allowing chemical modifications and thus fluorescent labeling. Then, the labeling can be performed by post-modification of commercially available CTA used in RAFT polymerization. York *et al.* synthesized poly(HPMA–DMAPMA) (poly(hydroxypropyl-dimethylaminopropyl)methacrylamide) by RAFT polymerization (Fig. 31).^79^ Although the copolymer chains were terminated by COOH functions, the authors chose to modify the thiocarbonylthio moiety. After polymerization, the dithioate ester end group of CTA was first reduced using NaBH_4_ and then reacted with cystamine to provide a free amine. Finally, fluorescent labeling was performed between the free amino function and NHS-fluorescein (5-SFX, Fig. 4B) by amide coupling.

**Fig. 32:**
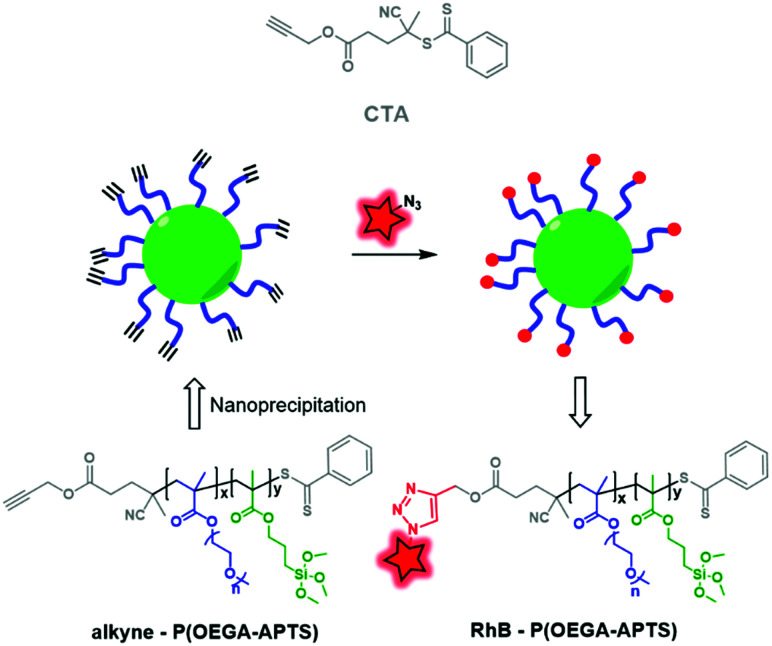
P(OEGA–APTS) BCP obtained by RAFT polymerization using a clickable CTA, allowing labeling by RhB-azide.

The second approach consists in synthesizing a CTA that already possesses a functionalizable site. Müller and coworkers developed clickable hybrid NPs based on a brush-like copolymer by designing a CTA bearing a clickable moiety.^81^ A hydrophilic block, POEGA (poly(oligo ethylene glycol)acrylate), and a hydrophobic one, PAPTS (poly(3-acryloxylpropyl)trimethoxysilane), were copolymerized by RAFT polymerization using propargyl ((4-cyanopentanoic acid)-4-dithiobenzoate) as the CTA (Fig. 32). After formulation, the brush-like BCP led to NPs displaying alkyne functions that were successfully used to click rhodamine B-azide (Fig. 4A). This approach consisting in fluorescent labeling the NPs has opened up new possibilities for ligation using, for instance, bioconjugates to target cancer cell receptors.

**Fig. 33:**
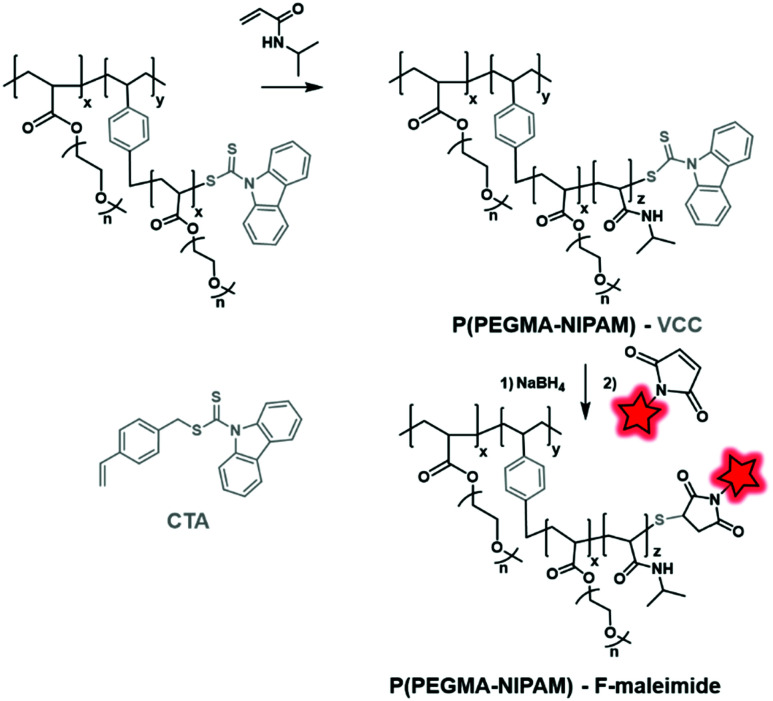
CTA reduction by NaBH_4_, followed by fluorescent labeling as described by Feijen *et al.*

Using this approach, Feijen *et al.* developed PEGMA–PNIPAM (poly(*N*-isopropylacrylamide)) using 4-vinylbenzyl 9*H*-carbazole-9-carbodithioate (VCC) as both the CTA and monomer (Fig. 33).^83^ The latter bears a vinyl function and a reductive carbodithioate moiety that can be reduced for post-labeling. After sequential RAFT polymerization reactions, the capping carbodithioate was reduced into thiol by NaBH_4_, thus allowing the labeling of the polymer by maleimide-fluorescein (Fig. 4B). The NPs obtained after the formulation of the labeled polymer were then used to study their cellular uptake by fluorescence imaging.

**Fig. 34:**
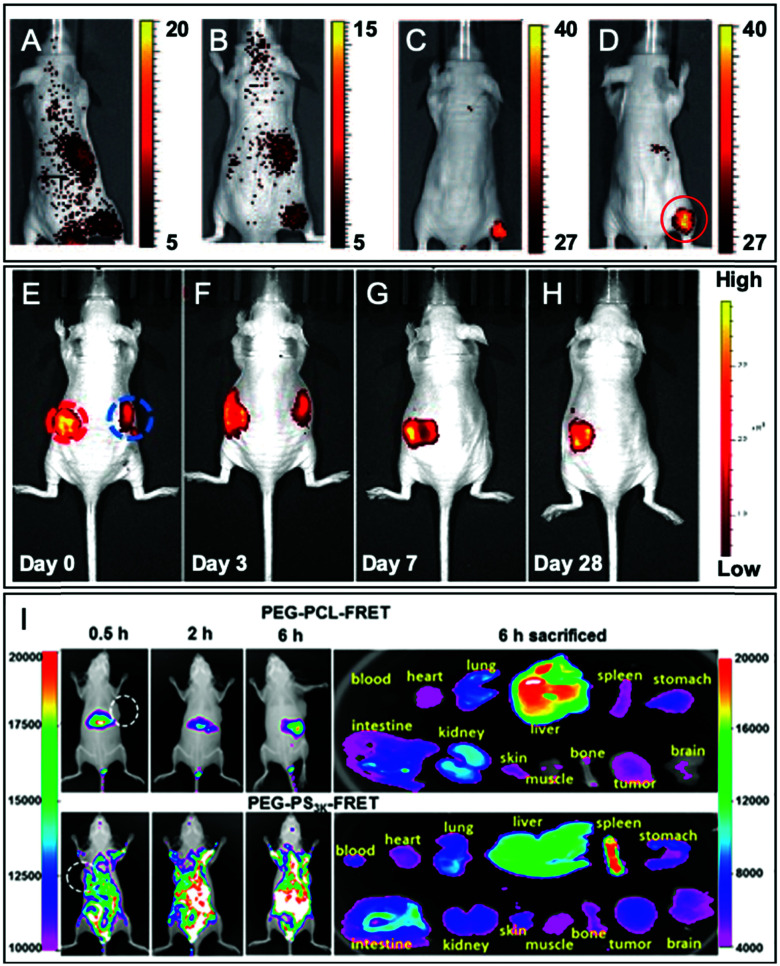
Examples of *in vivo* imaging using various fluorescent BCPs obtained by post-polymerization labeling. (A–D) Images of tumor-bearing nude mice, 24 h after injection with various fluorescently labeled amphiphilic BCPs (the tumor location is indicated by the red circle): non-functionalized NPs (A), NPs functionalized with folic acid (B), RGD peptide (C), and galactosamine (D). Adapted with permission from ref. 124. Copyright (2011) Wiley. (E–H) Fluorescence tracking after subcutaneous injection of NPs based on the rhodamine-conjugated BCP (left red circle) and rhodamine-encapsulated BCP (right blue circle). Adapted with permission from ref. 125. Copyright (2017) American Chemical Society. (I) Intravenous injection of fluorescent PEG–PCL and PEG–PS NPs in tumor-bearing mice and their biodistribution. Adapted with permission from ref. 60. Copyright (2018) American Chemical Society.

These examples show that, compared to polyesters, the labeling of polyacrylates at their terminal end involves several steps of post-modification or requires the development and synthesis of the adapted CTAs. Overall, the labeling of the terminal end limits the number of FMs per linear chain to two compared to backbone labeling. However, since both polyesters and polyacrylates possess different orthogonal functions at their terminal ends, the post-labeling by modification of the CTA can allow orthogonal terminal end labeling. This asset could lead to the design of FRET systems where the donor and the acceptor would be at each end of the copolymer.

## Conclusion

BCPs have attracted growing interest for the development of biocompatible nanomaterials owing to their well-controlled chemistry and to the versatility brought by the combination of various blocks with defined properties. The fluorescent labeling of these biocompatible materials appears to be a method of choice to understand their interactions in the fields of drug release, medicine and bioimaging. As described earlier, most of the presented fluorescent BCPs were formulated as polymeric NPs which were used in cell or *in vivo* imaging.^50,131^ Currently, the fluorescent labeling of BCPs is performed using two main methods: encapsulation in NPs and chemical ligation.^30^ Although encapsulation is a simple method, it presents a major drawback: the leakage of the FM out of the polymeric matrix. Consequently, the covalent fluorescent labeling of the BCP circumvents this issue. Compared to the encapsulation approach, covalent fluorescent labeling is tedious, but recent advances confirm the interest in this method. In this review we first emphasized the importance of the choice of the fluorophore, which has to be chosen considering its photophysical properties, its chemical stability, and the chemical ligation that it can enable.

In the second part we focused on the different methods to fluorescently label BCPs covalently. The first approach consists in the direct introduction of a fluorescent label in the BCP during the polymerization process. This can be achieved by the modification of the fluorophores into initiators or monomers through the addition of a specific function like hydroxyl, vinyl, allyl, or methacrylate.^52,95,110^ This approach is appealing as it directly furnishes a fluorescently labeled BCP in one step. However, several drawbacks should be pointed out. First, this method requires fluorescent precursors or monomers that are currently not easily available on the market and thus need to be synthesized. Moreover, during the polymerization step the FM can be exposed to harsh conditions (high temperatures, extreme pH), leading to its partial or total degradation with significant loss of its photophysical properties. Additionally, in some reported cases, the FM can lower the efficiency of the polymerization process.

The second approach consists in labeling the BCP after the polymerization step. This approach circumvents the aforementioned drawbacks in some aspects as the chemical ligation often proceeds in milder conditions than those required for polymerization. Moreover, the chemistry that is generally involved in post-modification is allowed by the use of a commercially available FM.

Notable differences of strategies arose from the nature of the BCP. Unlike polyacrylates, polyesters (including PLA, PLGA, and PCL) rarely display reactive functions in their backbone and are thus labeled at their extremity. Conversely, polyacrylates generally possess functions all along their backbone and the labeling of their terminal end requires chemical modification or development of new CTA displaying amine, alkyne, and thiol functions.^74,76,78^ Interestingly, the dye location and the dye content can be adapted considering the targeted applications. This advantage could also help to develop FRET systems within the same polymer to study their folding, stability or structural evolution in biological media. Finally, the choice of the labeling strategy can impact the bioimaging applications as it will define the aggregation properties leading to AIE or ACQ phenomena. It is also important to note that a high dye loading does not necessarily lead to bright polymeric NPs due to the ACQ phenomenon that lowers their quantum yield. Interestingly, this issue can be easily circumvented by mixing the labeled polymer with its non-labeled cognate prior to formulation in order to dilute and thus “de-aggregate” the FMs. Also, special attention should be paid to the nature of the covalent bond between FMs and copolymers which is determined by the choice of labeling strategy. More specifically, ester bonds can be prematurely cleaved by the action of enzymes (esterases), thus leading to a loss of brightness and the increase of the background noise in bioimaging.

In conclusion this review intends to guide chemists willing to fluorescently label biocompatible block copolymers by reviewing both FM and the methods used for ligation. As highlighted, these methods present numerous advantages to control and ensure efficient fluorescent labeling in the field of bioimaging. As perspectives, we believe that the continuous and convergent efforts being made to develop both FMs (and their availability on the market) and the labeling methods will certainly lead to brighter BCP-based nanomaterials that will find use in advanced bioimaging applications.

## Conflicts of interest

There are no conflicts to declare.
